# m6A and m5C modification of GPX4 facilitates anticancer immunity via STING activation

**DOI:** 10.1038/s41419-023-06241-w

**Published:** 2023-12-08

**Authors:** Baoxiang Chen, Yuntian Hong, Xiang Zhai, Yanrong Deng, Heng Hu, Shunhua Tian, Yukang Zhang, Xianghai Ren, Jianhong Zhao, Congqing Jiang

**Affiliations:** 1 Department of Colorectal and Anal Surgery, Zhongnan Hospital of Wuhan University, Wuhan, 430071 China; 2 Hubei Key Laboratory of Intestinal and Colorectal Diseases, Zhongnan Hospital of Wuhan University, Wuhan, 430071 China; 3 Clinical Center of Intestinal and Colorectal Diseases of Hubei Province, Zhongnan Hospital of Wuhan University, Wuhan, 430071 China; 4 Rosalind & Morris Goodman Cancer Institute, McGill University, Montreal, QC H3G 0B1 Canada

**Keywords:** Gastrointestinal cancer, Chemokines, Cancer microenvironment

## Abstract

Cancer immunotherapy is arguably the most rapidly advancing realm of cancer treatment. Glutathione peroxidase 4 (GPX4) has emerged as the vital enzyme to prevent lipid peroxidation and maintain cellular redox homeostasis. However, the mechanism of GPX4 in the regulation of cancer immunotherapy of colon adenocarcinoma (COAD) are incompletely understood. In pan-cancer analysis, we found that GPX4 showed remarkably upregulated expression and exhibited significant association with overall survival in multiple cancer types, especially COAD. Furthermore, upregulated GPX4 expression was positively correlated with increased immune cells infiltration and enhanced expression of immunomodulators. Mechanistically, *RBM15B*- and *IGFBP2*-mediated N6-methyladenosine (m6A) modification and *NSUN5*-mediated 5-methylcytosine (m5C) modification of GPX4 facilitated anticancer immunity via activation of cyclic GMP-AMP synthase (cGAS)-stimulator of interferon (STING) signaling by maintaining redox homeostasis in COAD. The risk model and nomogram model constructed based on the GPX4-derived genes further confirmed the prognostic and treatment-guiding value of GPX4. In all, our study demonstrated that m6A and m5C modification of GPX4 may be a promising target for cancer immunotherapy via activating the cGAS-STING signaling pathway in COAD.

## Introduction

Colon adenocarcinoma (COAD) is one of the most common cancers of gastrointestinal tract and has become the third leading cause of cancer-related mortality worldwide [1]. Complete surgical resection remains the primary curative treatment for non-metastatic COAD, whereas chemoradiotherapy combined with molecular-targeted therapy is preferred for advanced COAD [2]. Nevertheless, the outcome of conventional treatments for advanced metastatic COAD remains less favorable and the responses are brief [3]. Furthermore, most cases are diagnosed at an advanced stage (stage III-IV), and the 5-year survival rate of patients with advanced COAD with distant metastases is only approximately 10% [3]. Therefore, gaining comprehensive insights into the pivotal mechanisms and biological processes of tumorigenesis and the development of COAD are of importance.

Cancer immunotherapy aims to control multi-malignant phenotypes by activating the immune system to attack cancer cells. This has shown tremendous promise for cancer therapy, especially in solid and hematologic tumors [4]. Immune checkpoint inhibitors (ICIs), such as anti-PD-1, anti-PD-L1, and anti-CTLA-4, have revolutionized the therapeutic landscape and have shown better clinical efficacy in patients with MSI-H/dMMR COAD [5]. Notably, 85% of patients harboring COAD with MSS, called immune deserts—the so-called immune “cold” tumors—exhibit poor response to ICIs therapy [5–7]. However, only 30–50% of patients with COAD with MSI-H/dMMR benefit from ICI treatment, and 27% of patients with pMMR COAD achieve pathological responses, including three major pathological responses and one partial response to ICI combination therapy (anti-PD-1 + anti-CTLA-4) [8, 9]. These wide ranges of clinical findings led us to investigate effective biomarkers that can accurately differentiate between “cold” and “hot” tumors. Also, more potent synergistic therapeutic options that can robustly transform immunologically “cold” tumors into “hot” tumors are extremely in demand, with the intent of improving COAD immunotherapy outcomes.

Glutathione peroxidase 4 (GPX4), an antioxidant enzyme with pleiotropic functions, has been demonstrated to be an essential regulator of ferroptotic cell death [10, 11]. During catalysis, GPX4 removes accumulated intracellular lipid peroxides to maintain redox homeostasis [12]. Recently, it was confirmed that GPX4 inactivating agent not only inhibits triple negative breast cancer (TNBC) via promoting ferroptosis, but also reshapes the tumor microenvironment, thus turning the “cold” tumor of LAR-type TNBC into a “hot” tumor [13]. Combination therapy using GPX4 inhibitors and immunotherapy can further activate T cell infiltration in the tumor microenvironment (TME), and its clinical efficacy is significantly superior to that of monotherapy, indicating its potential as a novel precision medicine strategy in LAR-type TNBC [13]. cGAS-STING signaling is a cytosolic DNA-sensing pathway that activates the expression of type I interferons (IFN), pro-inflammatory cytokines, and chemokines during anticancer immune response [14]. In pancreatic ductal adenocarcinoma, a high-iron diet or GPX4 depletion promotes 8-OHG release, thereby activating the cyclic GMP-AMP synthase (cGAS)-stimulator of interferon (STING) pathway and resulting in macrophage infiltration and activation [15].

Therefore, in this study, we performed pan-cancer analysis and characterized the immune infiltration patterns with respect to GPX4 expression. We elucidated the novel mechanism of GPX4 action in regulating STING-mediated interferon signaling in COAD. Our results demonstrated that N6-methyladenosine (m6A) and 5-methylcytosine (m5C) modification of GPX4 activates STING by maintaining redox homeostasis during cancer immunotherapy. These data indicated that GPX4 is an innovative target for cancer immunotherapy in patients with COAD.

## Materials and methods

### Pan-cancer analyses

The RNA sequencing expression profile (reflected as FPKM value), copy number variation (CNV) data, somatic mutation profile [Mutation Annotation Format], and clinical data for 33 types of human cancer were acquired from The Cancer Genome Atlas (TCGA) via the Genomic Data Commons (GDC) data portal. The abbreviations for the cancer types are expanded and listed in Supplementary Table 1. GPX4 expression in various cancer types was evaluated using the Tumor Immune Estimation Resource (TIMER) web server. Genomic alterations (amplification or deletion) were analyzed by GISTIC 2.0 using CNV data. MSI analysis was performed based on the study of Bonneville [16]. Tumor mutational burden (TMB) data were processed using VarScan2 and analyzed in clusters using the maftools package, whereas methylation data were obtained from the LinkedOmics data portal.

### Collection of COAD datasets

The GSE17538 datasets were downloaded from the NCBI Gene Expression Omnibus (GEO). The downloaded Affymetrix CEL files were background-corrected, and quantile normalized using the Bioconductor Affy and SimpleAffy packages. For the other normalized datasets, microarray expression files were curated directly for profiling.

### Estimation of immunological features

Immunological analysis was performed as previously described, with minor adjustments [17]. Immunological characteristics, including checkpoint genes, immunomodulators, tumor-infiltrating lymphocytes, and HLA, were calculated using the ssGSEA algorithm derived from the GSVA package and the corresponding gene sets. A series of specific gene sets associated with known biological processes, including immune checkpoints, effector CD8 + T cells, DNA damage repair, Pan-F-TBRS, EMT1-3, antigen processing machinery, mismatch repair, and DNA replication, were obtained from published literature [18]. The activities of these biological processes were quantified using the ssGSEA algorithm. Immune, stromal, and estimate scores were calculated using the ESTIMATE algorithm according to the expression data. Furthermore, the activities of various processes in the cancer immunity cycle were estimated using ssGSEA based on individual gene expression profiles.

### Construction of the GPX4-derived genomic model

The GPX4-derived genes were identified from the COAD cohort of TCGA by Spearman analysis, with cut-offs of |r | > 0.6 and *P* < 0.01. Then prognostic GPX4-derived genes were identified using univariate Cox regression analysis with *P* < 0.05 by the survival R package. The selected prognostic genes were used as inputs for random forest (RF) analysis, and the most important GPX4-derived genes were identified using the RF R package. These genes were included in the multivariate-Cox regression analysis, and the GPX4-derived genomic model was constructed using the survival R package and the following formula:

$${\rm{risk\; score}}=\Sigma ({\rm{Coefi}}\times {\rm{Expi}}),$$

Where Expi represents the expression of signature genes and Coefi represents the risk coefficient. Patients with COAD were further divided into high- and low-risk subgroups based on the mean risk score.

### Functional enrichment analysis

Gene set enrichment analysis (GSEA) was conducted to investigate the disparity of signaling pathways activated in two distinct subpopulations. Gene Ontology (GO) enrichment and Kyoto Encyclopedia of Genes and Genomes (KEGG) pathway analyses were conducted using the clusterProfiler R package. The activities of hallmark gene sets obtained from the Molecular Signatures Database were quantified using ssGSEA.

### Mutation analysis and drug susceptibility estimation

Mutation waterfall plots were visualized using the Bioconductor maftools R package. The correlation between GPX4 expression and anticancer drug sensitivity was evaluated using the Genomics of Drug Sensitivity in Cancer (GDSC) dataset, a large-scale cancer cell line-based dataset having data on response to anticancer agents. Next, the half-maximal inhibitory concentration (IC50) was calculated to represent the drug response using the pRRophetic R package.

### Nomogram establishment and validation

The associations between clinicopathological indicators and GPX4-derived risk scores with OS were evaluated using univariate and multivariate-Cox regression models. Then, the predictive nomogram was established and calibration curves were plotted using the RMS R package. Decision curve analysis (DCA) was used to evaluate clinical performance by quantifying net benefits at different threshold probabilities. Receiver operating characteristic (ROC) curves were drawn over time and used to estimate the predictive ability of this nomogram.

### Specimen collection and ethics approval

Fifty-four fresh COAD specimens and paired adjacent paracancerous tissues were obtained in a blinded manner from patients undergoing surgery at the Zhongnan Hospital of Wuhan University. The detailed clinical and pathological features of the study population are listed in Supplementary Table 2. Informed written consent was obtained from all the participants. This study was approved by the Ethics Committee of the Zhongnan Hospital of Wuhan University.

### Cell culture and transfection

COAD cell lines (SW480 and HT29) were obtained from ATCC and cultured as previously described [19]. Transfections were performed with Lipofectamine 3000 (Invitrogen, Carlsbad, CA, USA) following the standard protocol.

### RNA isolation and real-time PCR (RT-qPCR)

RNA extraction, reverse transcription, and RT-qPCR were conducted as previously described [16, 19]. The sequences of specific forward and reverse primer used in this study are listed in Supplementary Table 3.

### Western blot analysis

Western blotting assays were conducted as previously described [19]. Primary and secondary antibodies used are listed in Supplementary Table 4.

### Generation of GPX4 overexpression or knockdown cell lines

COAD cell lines stably overexpressing GPX4 were constructed by lentiviral transduction using the pCDH vector. The small hairpin RNA (shRNA)-mediated approach was used to screen GPX4 knockdown COAD cell lines. The sequences were listed in Supplementary Table 5. The overexpression or shRNA vector was co-transfected with the lentiviral packaging plasmids psPAX2 and pMD2.G into HEK-293T cells. Culture supernatants were harvested after 48 h and used to transfect COAD cells. The transfected cells were subjected to puromycin screening.

### Enzyme-linked immunosorbent assay (ELISA)

Cytokines or compounds were analyzed using ELISA following the manufacturer’s instructions. Commercial ELISA kits for IFN-β (BioLegend), 4-HNE (MyBioSource), and MDA (BioVision) were used for quantitative analysis.

### Luciferase reporter assay

Luciferase activity was determined using the Dual-Luciferase Reporter Assay System (Promega) as described previously [19]. Relative luciferase activity was estimated by normalizing the firefly luciferase activity to that of Renilla luciferase.

### Immunofluorescence analysis

COAD cells subjected to different treatments were plated onto confocal dishes, fixed, and incubated with primary antibodies overnight. After washing with PBS, the appropriate secondary antibodies were added, and the nuclei were counterstained with Hoechst 33342. Immunofluorescence images were captured using a laser-scanning confocal microscope (Leica, Germany).

### Animal model

Animal studies were approved by the Institutional Animal Care and Use Committee. The 6–8-week-old female C57BL/6 mice were kept at the Wuhan Institute of Virology, Chinese Academy of Sciences (Wuhan, China). The animals were randomly divided into two groups (12 mice/group). To develop the syngeneic model, 1 × 10^6^ MC38-*Ctrl* or MC38-*Gpx4*^KD^ cells were injected subcutaneously into the right flank of C57BL/6 mice, respectively. These two groups of mice were randomly and blindly allocated into separate groups 12 days after cell injection. Subsequently, either an anti-PD-1 antibody (5 mg/kg per mouse) or an IgG isotype was administered intraperitoneally every 2 days until the conclusion of the observation period. Subcutaneous tumor volumes (mm^3^) were estimated using the formula: ${\rm{volume}}=1/2\times {\rm{length}}\times {{\rm{width}}}^{2}$.

### Isolation of tumor infiltrating lymphocytes and flow cytometry analysis

In brief, the MC38 tumor tissues were dissected and prepared via mechanical disruption followed by collagenase P (Sigma-Aldrich, USA) and DNase I (Sigma-Aldrich, USA) at 37°C for 15 min. For surface staining, live cells were suspended in PBS and subsequently incubated with fluorochrome-conjugated antibodies: anti-CD45-PE (Biolegend), anti-CD3-FITC (Biolegend), or anti-CD8-PE (Biolegend). Intracellular staining was performed with anti-IFN-β-APC antibody (Biolegend) and TNF-α (Biolegend) after fixation and permeabilization using the Cytofix/Cytoperm Fixation /Permeabilization kit (BD Biosciences, USA). All stained cells were applied onto FACScan (Becton Dickinson, USA) and analyzed by Flowjo software (BD Biosciences, CA, USA).

### RNA stability assay

To analyze *GPX4* mRNA stability, stable transfected COAD cells were treated with actinomycin D (5 μg/ml) for the indicated times. The treated cells were then collected, and total RNA was extracted followed by RT-qPCR. The mRNA levels were normalized to their 0 h expression levels.

### Statistical analysis

All statistical analyses were conducted using R version 4.1.2 with appropriate packages; results with *P* < 0.05 indicating statistical significance. Normally distributed continuous variables were compared using the independent Student’s *t*-test or the Mann–Whitney U test. For comparison of categorical variables, chi-square or Fisher’s exact tests were applied. The Spearman rank correlation test was used to assess interactions between GPX4 expression, risk scores, and immune infiltration-related indices. The Kaplan–Meier method was used for generating survival curves, and log-rank tests were performed for prognostic analysis. Univariate and multivariate-Cox regression analyses were performed using the survival and forest plot packages in R. The clinical response to ICB therapy was predicted using the tumor immune dysfunction and exclusion (TIDE) algorithm. The discriminative value of the predictors was evaluated using the area under the curve (AUC) based on the time-dependent ROC curve analysis computed using the time ROC R package.

## Results

### GPX4 expression pattern, its prognostic significance, and immunological correlation in pan-cancer analysis

**Fig. 1:**
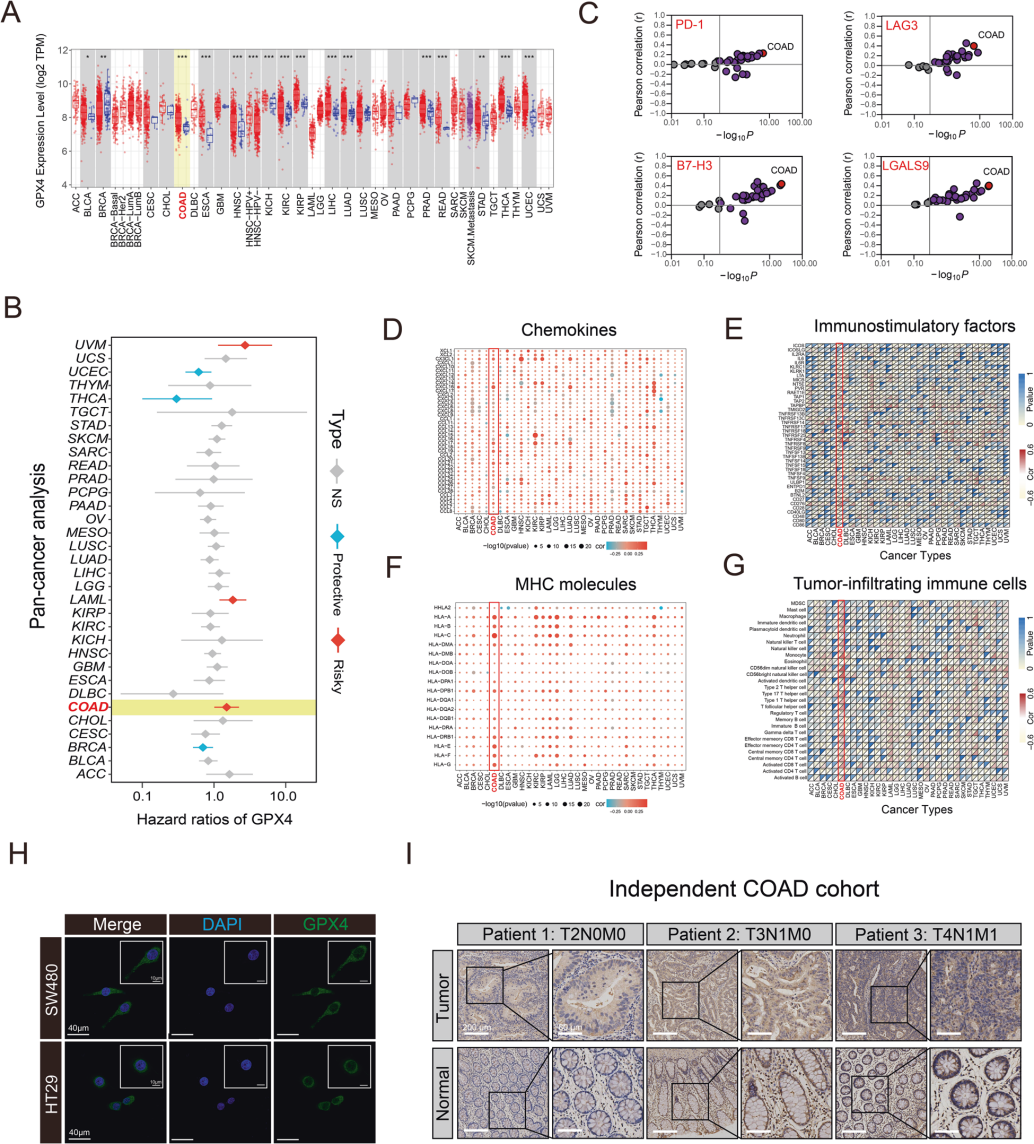
Pan-cancer expression pattern, prognostic significance, and immunological correlation of GPX4. **A** The expression levels of GPX4 in pan-cancer analysis using *TIMER* database. Blue dots represent normal tissues. Red Blue dots represent cancer tissues. Purple dots represent metastatic SKCM. **B** Risk plot of correlation between GPX4 with OS in pan-cancer analysis. **C** Analysis of the correlation between GPX4 expression and common immune checkpoints (PD-1, LAG3, B7-H3 and LGALS9). The dots represent cancer types. The red dot represents COAD. The Y-axis represents the Pearson correlation, while the X-axis represents -log_10_*P*. The purple dots represent statistically relevant cancer types. **D**–**G** The heatmaps showing the association of GPX4 with chemokines, immunostimulatory factors, MHC molecules, and tumor-infiltrating immune cells across pan-cancer types. **H** Immunofluorescence localization of GPX4 in COAD cells. **I** Immunohistochemistry analysis of GPX4 in carcinoma tissue and para-cancer (normal) of COAD patients. *P < 0.05; **P < 0.01; ***P < 0.001.

Comprehensive analysis of the TCGA and Oncomine databases revealed that GPX4 was more highly expressed in most cancers, such as COAD, STAD, LIHC, ESCA, and KIRC, than in normal tissues (Fig. 1A, Fig. S1A). In 54 pairs of COAD and normal tissues, GPX4 was more significantly expressed in cancer tissues than in the normal tissues (Fig. S1B). Next, we investigated the relationship between GPX4 expression and clinical outcomes. A diverse cohort of patients with 33 tumor types was grouped into high- and low-expression groups according to the median GPX4 expression. Survival analysis revealed notable differences between GPX4 high- and low-expression groups for several cancer types; patients with high-GPX4 expression levels had significantly shorter OS than those with low-GPX4 expression in the TCGA-COAD cohort (Fig. 1B). These results suggested that GPX4 could be developed as a potential prognostic cancer biomarker. In COAD, GPX4 expression was strongly positively correlated with several common immune checkpoints, including PD-1, LAG-3, B7-H3, and LGALS9 (Fig. 1C). This led us to explore the potential mechanisms underlying GPX4 involvement in cancer immunotherapy. As expected, the GPX4 expression was positively associated with most immunomodulators (chemokines, immunostimulatory factors, and MHC molecules) in COAD (Fig. 1D-F). We used the ssGSEA algorithm to estimate the abundance of Tumor-Infiltrating Immune Cells (TIICs) in the TME, and the results demonstrated GPX4 was positively associated with most TIICs in patients with COAD (Fig. 1G). MSI occurs because of the accumulation of insertion-deletion mutations in short repetitive DNA sequences caused by defects in DNA mismatch repair (MMR) [20]. GPX4 was positively associated with TMB and MSI in several cancers, indicating that it may reflect potential benefit from antitumor immunotherapy in these cancers (Fig. S1C, D). In addition, GPX4 was positively correlated to mismatch repair genes (*MLH1*, *MSH2*, *MSH6, PMS2*, and *EPCAM*) across cancer types (Fig. S1E). Upon performing an immunofluorescence assay, the GPX4 protein was seen to be mainly localized in the cytoplasm of COAD cells (SW480 and HT29) (Fig. 1H). Moreover, immunohistochemical staining indicated that the expression level of the GPX4 protein in COAD tissues was significantly increased compared with that in adjacent tissues (Fig. 1I and Fig. S1F, G). Taken together, the high expression pattern of GPX4 was TME-specific, and emphasized the clinical utility of GPX4 as a predictive biomarker in cancer immunotherapy.

### Immunological characteristics of GPX4 in COAD

**Fig. 2:**
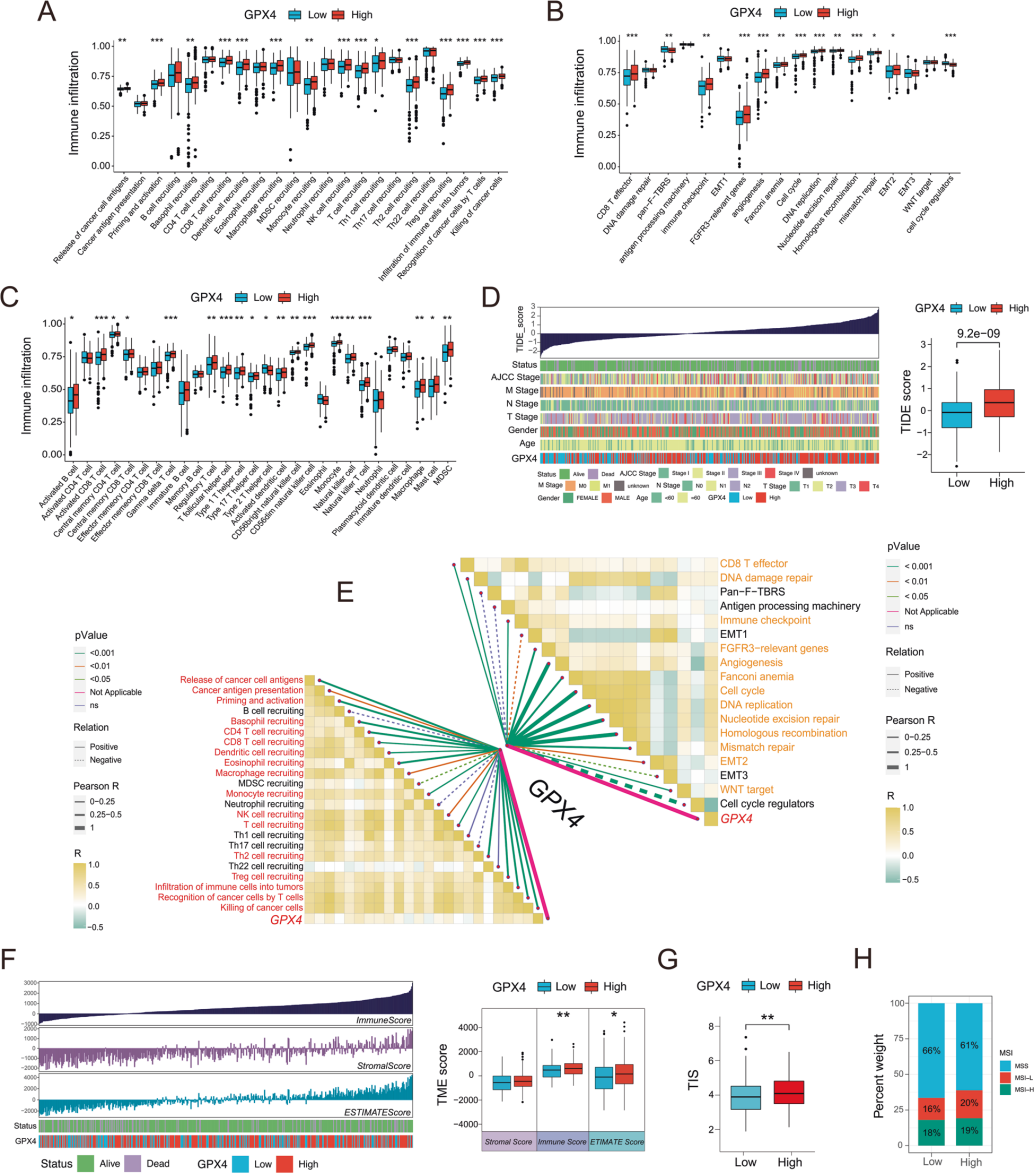
Immunological characteristics of GPX4 in COAD. **A** Quantification analysis of the activities of cancer immunity cycle in low and high GPX4 subgroups in COAD. **B** Comparisons of the activation of known biological signatures in low and high GPX4 subgroups. **C** The comparison of immune cells infiltration between GPX4 low- and high-expression subgroups. **D** The comparison of the TIDE scores between GPX4 low- and high-expression subgroups. **E** Spearman’s correlation analysis of the GPX4 expression with the activities of cancer immunity cycles (left) and known biological signatures (right) in TCGA-COAD. **F** Evaluation of Stromal, Immune and Estimate Score between GPX4 low- and high-expression subpopulations. **G** Quantification analysis of TIS in GPX4 low and high-expression subgroups. **H** The relationship between the expression of *GPX4* and MSI. *P < 0.05; **P < 0.01; ***P < 0.001.

**Fig. 3:**
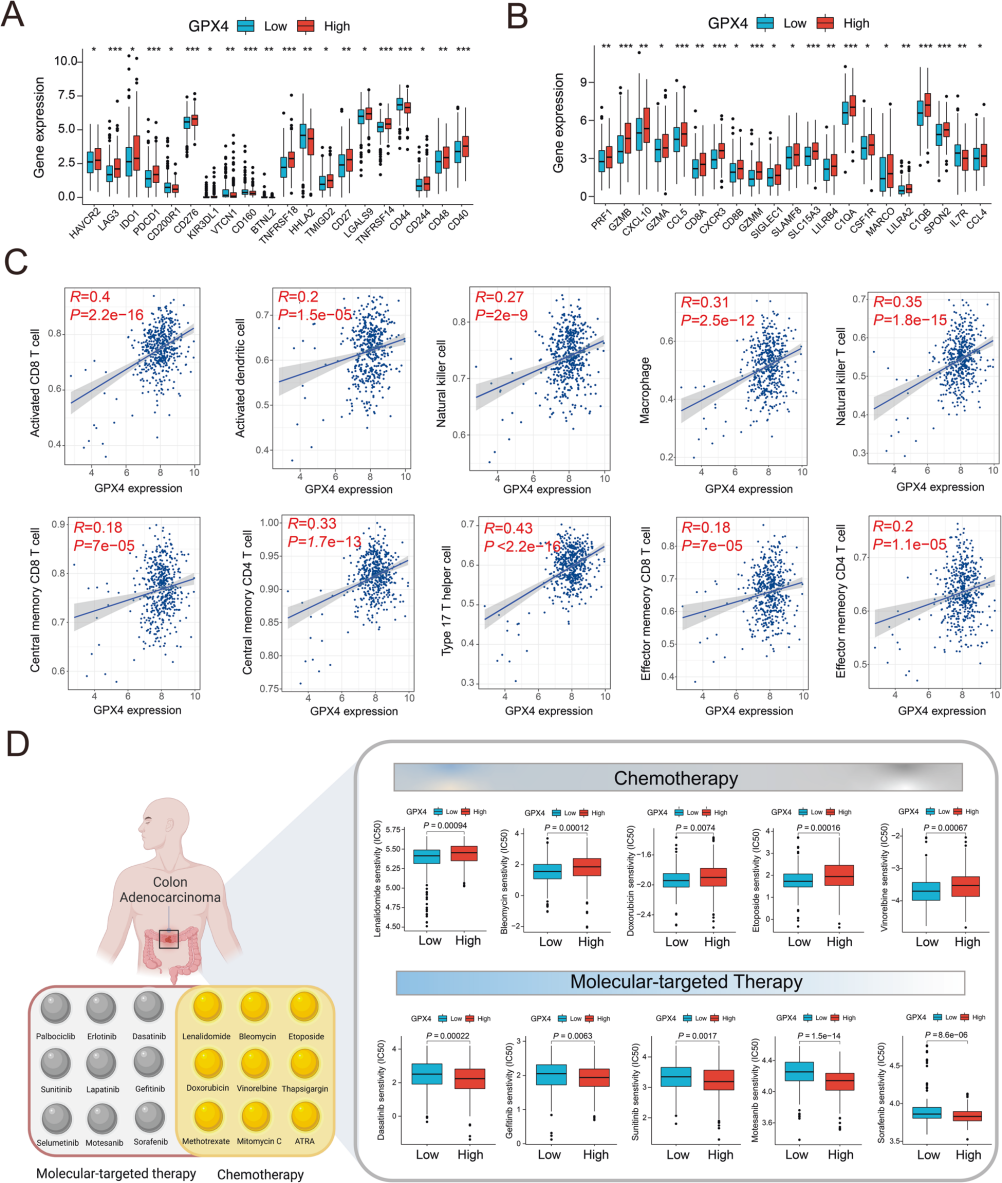
The correlation analysis between immune response and drug sensitivity with GPX4 expression. **A** The comparison of common immune checkpoints in GPX4 low- and high-expression subpopulations in COAD. **B** The comparison of common immune effector genes in GPX4 low- and high-expression subgroup. **C** The correlation analysis between GPX4 expression and immune cell infiltration in COAD. **D** The correlation analysis between GPX4 expression and several common chemotherapy drugs and molecular-targeted agents.

Next, immunological characteristics and potential mechanisms of GPX4 action in COAD were explored. Based on median GPX4 expression, TCGA-COAD cohort samples were divided into high- and low-GPX4 subpopulations. In the high-GPX4 expression subgroup, the activities of most steps within the cancer immunity cycle were significantly higher than those of the low-GPX4 subgroup (Fig. 2A). Also, we confirmed the relatively high activity of immune activation processes (such as immune checkpoints and effector CD8 + T cells) and stromal activation processes (such as angiogenesis and expression of FGFR3-related genes) in the GPX4 high-subgroup (Fig. 2B). Consistent with the results of the correlation analysis, a marked infiltration of immune cells associated with cancer immunotherapy, including CD8 + T cells, was observed in the GPX4 high-subgroup (Fig. 2C). Furthermore, the TCGA-COAD cohort with high GPX4 expression exhibited significantly higher TIDE scores (Fig. 2D). Moreover, high GPX4 expression was associated with increased immune and ESTIMATE scores in patients with COAD (Fig. 2F). Interactions between GPX4 and several biological processes and the cancer immune cycle were evaluated in the TCGA-COAD cohort. As depicted in Fig. 2E, GPX4 expression was strongly related to most steps of the immunity cycle, as well as the stromal and immune activation processes. In addition, our analysis exhibited higher T cell inflamed scores (TIS) in the GPX4-high subgroup than that of the GPX-low subgroup (Fig. 2G). Furthermore, patients with MSS COAD accounted for a higher proportion of the GPX4-low subpopulation (Fig. 2H). Common immune checkpoint genes, including *LAG3*, *IDO1*, *PDCD1*, and *HHLA2*, and many immune effector genes, such as *CCL4*, *CCL5*, *CXCL10*, *GZMA*, *GZMB*, *CXCR3*, and *IL7R*, were all highly expressed in the high-GPX4 subgroup (Fig. 3A, B). In line with previous results, the expression of GPX4 was significantly positively correlated with the infiltration level of activated CD8 + T cells, central memory CD8 + T cells, central memory CD4 + T cells, natural killer T cells, effector memory CD8 + T cells, effector memory CD4 + T cells, and other immune cells in COAD (Fig. 3C and S2A). The drug susceptibility analysis demonstrated that the low-GPX4 subpopulation exhibited a higher level of sensitivity to common chemotherapeutic drugs, such as bleomycin, doxorubicin, vinorelbine, and methotrexate, whereas the high-GPX4 subgroup showed a higher level of sensitivity to several targeted drugs, including gefitinib, bexarotene, motesanib, sorafenib, and lapatinib in COAD (Figs. 3D and S2B). As described in Fig. S3A and S3B, the high GPX4 expression was significantly correlated with antigen processing and presentation, as well as with the interferon response signaling pathways. Collectively, these analyses suggested that GPX4 is associated with cancer immunotherapy in patients with COAD.

### Establishment and verification of the GPX4-derived genomic model

To further explore the biological function of GPX4, 1224 GPX4-derived genes were identified in TCGA-COAD cohort by Spearman analysis, with cut-offs of |r | > 0.6 and *P* < 0.01. These GPX4-derived genes may play multiple roles in the metabolic reprogramming of COAD cells (Fig. S4A, B). Total 76 prognosis-related genes were identified in the COAD cohort using univariate Cox regression analysis. Supervised RF models were used to identify the most important GPX4-derived genes from these 76 prognosis-related genes. The RF-OOB algorithm selected seven GPX4-derived genes with minimal OOB error values as optimal features (Fig. S4C). Finally, these GPX4-derived genes were included in the multivariate-Cox regression model to construct the GPX4-derived genomic model (Fig. S4D). GPX4-derived risk-scoring system was developed following the formula: risk score = (−0.4618412) * TRAP1 expression + 0.80820433 * SPR expression + (−0.7943614) * MAPKAPK3 expression + 0.60498621 * MAN1B1 expression + 0.8769671 * MTAP-007 expression (Fig. S4D). Based on the mean risk score value, patients with COAD were stratified into high- and low-risk subpopulations (Fig. S4E). Notably, significantly more patients with COAD died in the high-risk subgroup, and patients in the high-risk subgroup also exhibited worse OS, DSS, and PFS than those in the low-risk group (Fig. S4F and H–J). ROC analysis indicated that the GXP4-derived risk score possessed good potency and had favorable AUC for predicting 1-, 3-, and 5-year OS (Fig. S4G). The risk model constructed based on GPX4-derived genes was validated in an independent cohort (GSE17538) and the results suggested that the high-risk patients had poorer OS and DSS than those of low-risk patients, although PFS did not differ significantly (Fig. S4K–M). The top 20 mutated genes in the high and low-risk score groups of TCGA-COAD are shown in Fig. S5A, B. The mutation frequencies of *TP53*, *TTN*, and *KRAS* were remarkably different between these two groups. Univariate and multivariate-Cox regression analyses showed that this risk score model was an independent prognostic factor for COAD (Fig. S5C, D). These results suggest that the GPX4-derived genomic model could be a promising prognostic biomarker for patients with COAD.

### Construction and validation of COAD specific nomogram

To help clinicians with readable and quantitative measurements, a nomogram based on the GPX4-derived risk score was established to visually assess the survival outcomes of patients with COAD (Fig. S5E). The predictive performance of the nomogram was evaluated using ROC analysis and the calibration curve. In the ROC analysis, the AUC values for the 1-, 3-, and 5-year OS were 0.750, 0.762, and 0.766, respectively (Fig. S5F). The calibration plot confirmed 1-, 3-, and 5-year OS of the constructed nomogram with the actual probability (Fig. S5G). Furthermore, DCA revealed the maximum net clinical benefit and broadest threshold of the nomogram model than that of other models (Fig. S5H). Patients with COAD were stratified into low- and high-risk subpopulations based on the median risk score calculated using the GPX4-derived nomogram. Further survival analysis showed that high-risk patients with COAD exhibited markedly worse outcomes than those of the low-risk subgroup (Fig. S5I). Therefore, it can be concluded that the GPX4-derived nomogram possesses superior predictive capability in patients with COAD.

### Positive regulation of GPX4 in the cGAS-STING pathway

**Fig. 4:**
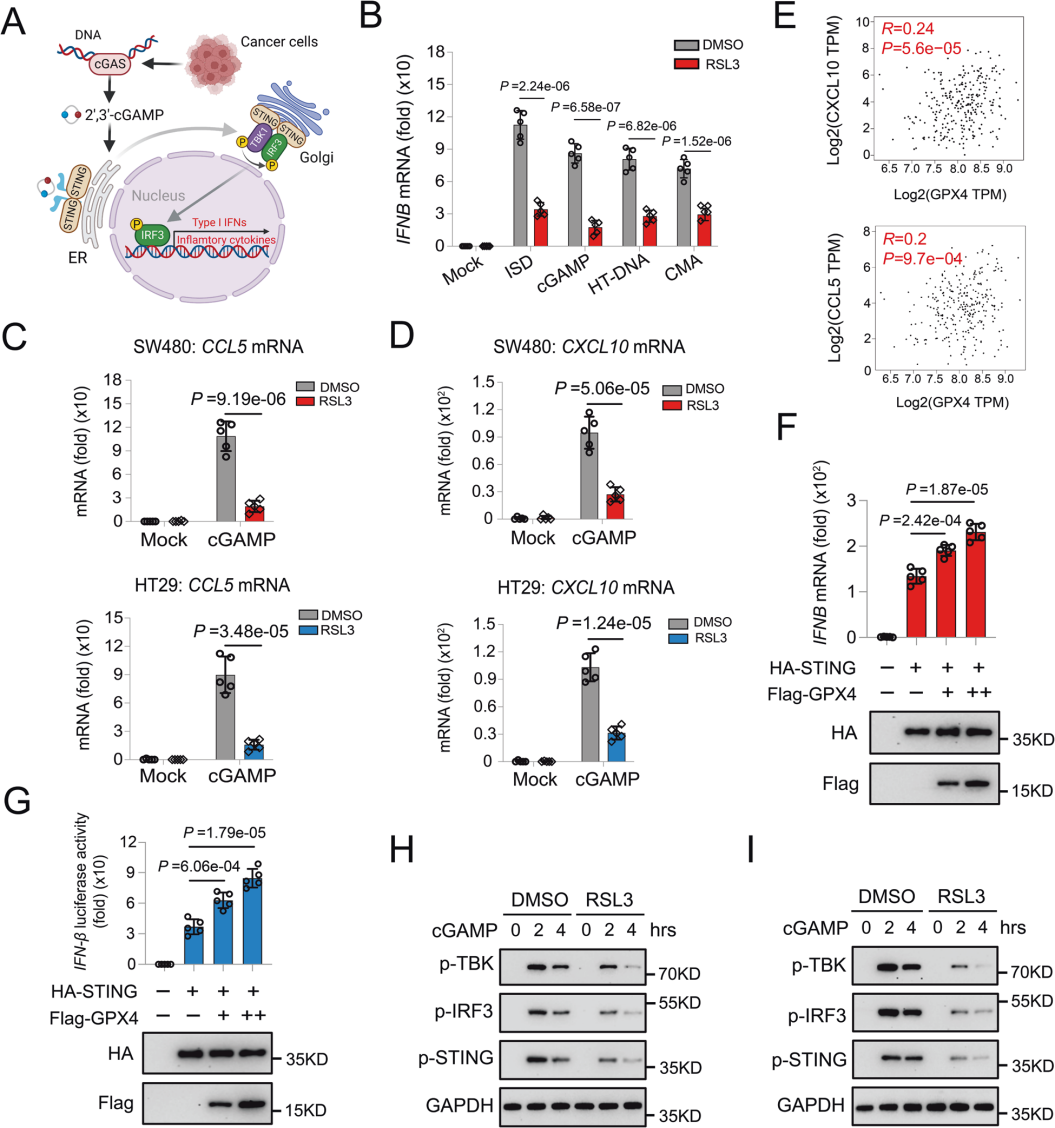
The positive regulation of GPX4 in the cGAS-STING pathway. **A** Schematic illustration of the cGAS-STING signaling pathway. **B** RT-qPCR analysis of the *IFNB* mRNA level in SW480 cells pretreated with DMSO or RSL3 and plus stimulation as indicated. **C**, **D** RT-qPCR analysis of the *CCL5* and *CXCL10* mRNA level in COAD cells pretreated with DMSO or RSL3, and then stimulated with cGAMP. **E** Correlation analysis of the expression of *GPX4* level with the *CXCL10* and *CCL5* expression using the *GEPIA* tool in TCGA-COAD. **F**, **G** The RT-qPCR analysis and luciferase reporter assays showing the relative *IFNB* mRNA expression and *IFN-β* luciferase activity in HEK-293T transfected with indicated plasmids. **H**, **I** Immunoblot analysis of phosphorylation of STING, TBK1 and IRF3 in the control and GPX4-knockdown COAD cells (SW480 and HT29).

DNA released from cancer cells or bacteria activates cytosolic cGAS to synthesize cyclic GMP-AMP (cGAMP), which combines with STING to form dimers [21, 22]. This results in TBK1 kinase recruitment, IRF3 activation, and induced secretion of type I interferon (IFNA) and pro-inflammatory cytokines that confer innate immunity (Fig. 4A) [21, 22]. Due to its robust ability to promote resistance against pathogen and clearance of malignant cells, the cGAS-STING signaling pathway has recently received considerable attention, especially in immunotherapy [21]. As is well known, activation of the cGAS-STING signaling pathway regulates intratumor infiltration of T cells and B cells [23, 24]. GPX4 inactivation leads to overwhelming lipid peroxidation and can regulate cGAS-STING signaling pathway in innate antiviral immune responses induced by HSV-1 [25]. Consequently, we investigated whether GPX4 activates cGAS-STING signaling pathway and induces immune cell infiltration in COAD independent of ferroptosis. RSL3 is a classical covalent inhibitor of GPX4, which binds directly to GPX4 and helps to accumulate lipid hydroperoxides [26]. In our cell viability assays, RSL3 did not reduce the viability of SW480 and HT29 cells at various doses (Fig. S6A, B). RSL3 administration potently inhibited *IFNB* mRNA expression induced by STING pathway agonists, including interferon-stimulating DNA (ISD), 10-carboxymethyl-9-acridanone (CMA), herring testes DNA (HT-DNA), and endogenous STING ligand cGAMP (Fig. 4B). In addition, FIN56 leads to a reduction in GPX4 protein abundance, and its treatment also significantly inhibits *IFNB* mRNA expression in CRC cells induced by the same activators (Fig. S6C). Based on these results, we further investigated whether GPX4 is involved in the positive regulation of cGAS-STING signaling pathway. RT-qPCR analysis indicated that the cGAMP-triggered expression of *CXCL10* and *CCL5* mRNAs were markedly decreased in COAD cell lines with RSL3 treatment (Fig. 4C, D). Notably, it was also demonstrated that the expression level of *GPX4* was significantly positively correlated with *CCL5*, *CXCL10*, *CXCL11*, *ISG15*, and *ISG56* in TCGA-COAD from GEPIA database (Fig. 4E and Fig. S6D–F). The luciferase reporter assay and RT-qPCR analysis further confirmed that GPX4 potentiated the activation of *IFNB* reporter and *IFNB* mRNA expression mediated by STING (Fig. 4F, G). Furthermore, GPX4 inhibition attenuated the phosphorylation of STING, TBK1, and IRF3 in SW480 and HT29 cells (Fig. 4H, I). Taken together, these results demonstrated that GPX4 positively regulates the cGAS-STING signaling pathway.

### GPX4 augments the cGAS-STING pathway through the inhibition of lipid peroxidation

**Fig. 5:**
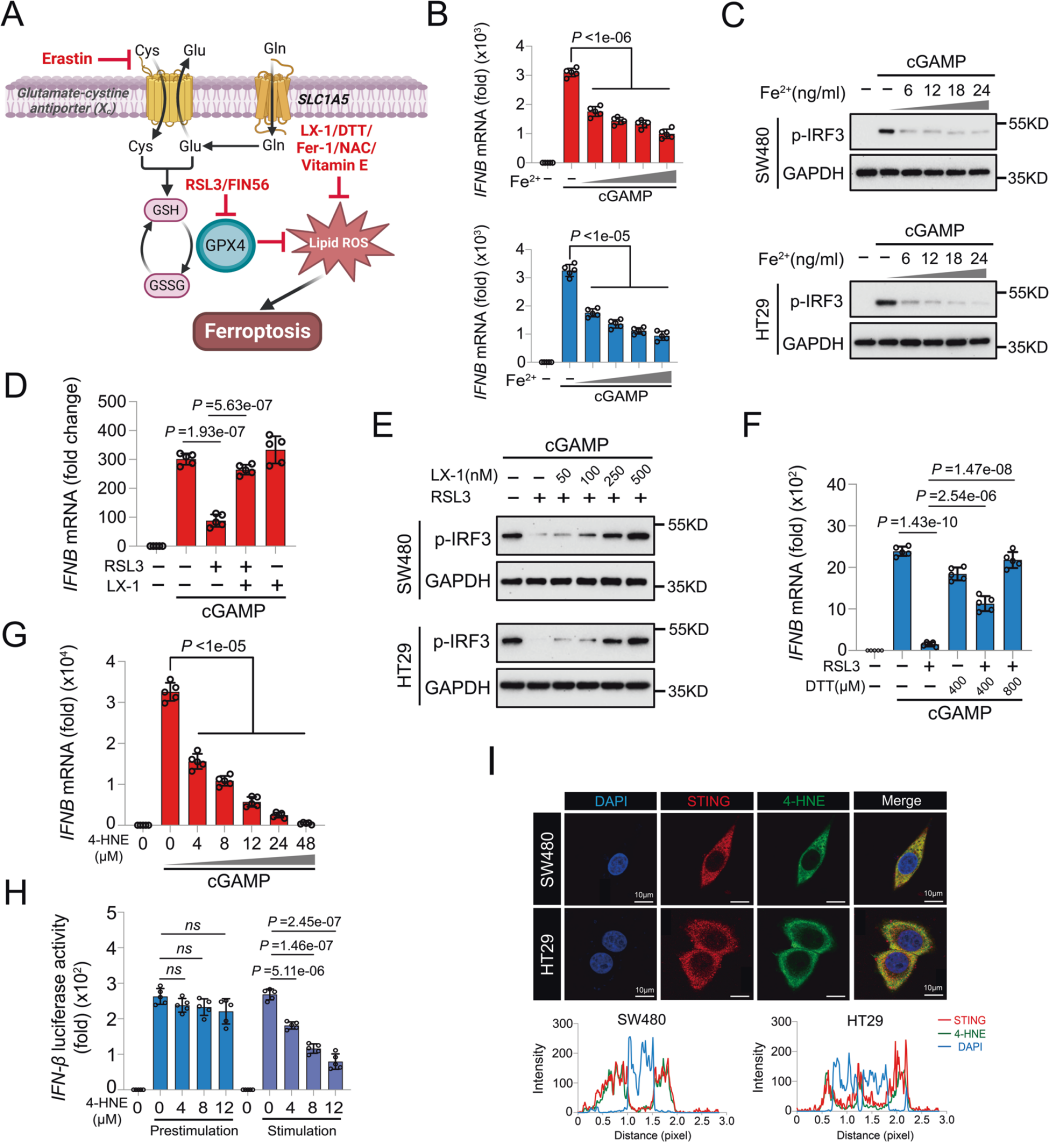
GPX4 augments the cGAS-STING pathway through the inhibition of lipid peroxidation. **A** Schematic representation of GPX4 inactivation leads to accumulation of lipid ROS and cell ferroptosis. **B** RT-qPCR analysis of *IFNB* mRNA level in SW480 (red) and HT29 (blue) cells pretreated with increasing Fe^2+^, followed by cGAMP treatment. **C** Immunoblot analysis of the phosphorylation of IRF3 levels in COAD cells retreated with increasing Fe^2+^ (0, 6, 12, 18, 24 μM) and cGAMP. **D** RT-qPCR analysis of *IFNB* mRNA level in SW480 cells with LX-1 and RSL3, followed by cGAMP treatment. **E** Immunoblot analysis of the phosphorylation of IRF3 levels in COAD cells pretreated with RSL3 and increasing concentrations of LX-1, followed by cGAMP treatment. **F** RT-qPCR analysis of *IFNB* mRNA level in HT29 cells with indicated concentration of DTT and 0.5 μM RSL3, followed with cGAMP stimulation. **G** RT-qPCR analysis of *IFNB* mRNA level in SW480 cells with increasing 4-HNE and then stimulated with cGAMP. **H** Luciferase reporter assays of *IFN-β* luciferase activity in HEK293T cells with increasing 4-HNE and different stimulation patterns. **I** Fluorescent staining of 4-HNE (green) and STING (red), and corresponding fluorescence intensity–QD concentrations curves in SW480 and HT29 cells, respectively.

The cystine/glutamate antiporter (System Xc-) transfers extracellular cysteine to cells, contributing to intracellular glutathione (GSH) synthesis. However, treatment with lipid peroxidation inhibitors, such as liproxstatin-1 (LX-1), vitamin E, dithiothreitol (DTT), and dithiothreitol, efficiently alleviates excessive cellular accumulation of lipid ROS (Fig. 5A) [25]. GPX4 uses GSH as the co-substrate to scavenge lipid peroxides, which in turn enhances the cGAS-STING signaling pathway in innate antiviral immune responses [25]. Ferrous ions (Fe^2+^) enhance ROS generation via Fenton reaction, resulting in the intracellular accumulation of lipid peroxides (Fig. 5A) [27]. RT-qPCR and western blot assays revealed that in COAD cell lines, Fe^2+^ significantly suppressed the expression of cGAMP-induced *IFNB* in a dose-dependent manner (Fig. 5B, C). GPX4 is essential for maintaining redox stability and suppressing lipid peroxide accumulation. Therefore, we confirmed the effect of lipid peroxidation inhibitors in reversing the inhibition of GPX4 inactivation in cGAS-STING pathway. Figure 5D, E have shown that the expression level of cGAMP-induced *IFNB* mRNA and the phosphorylation level of IRF3 in RSL3-treated COAD cells were gradually restored after the addition of LX-1. Likewise, RSL3-mediated GPX4 inhibition in the cGAS-STING pathway was reversed by pretreatment with other lipid peroxidation inhibitors, including DTT, vitamin E, and NAC (Fig. 5F and Fig. S6G, H). As the final product of cellular lipid peroxidation, 4-HNE was used to evaluate the potential role of cCAS-STING signaling in COAD. We observed a significant increase in 4-HNE levels in CRC cells following either RSL3 treatment or GPX4 knockdown (Fig. S6I). cGAMP-induced *IFNB* expression was markedly decreased in a dose-dependent manner following 4-HNE treatment (Fig. 5G). Notably, the inhibitory effect of 4-HNE on cGAMP-induced *IFNB* expression was not reversed by LX-1 treatment (Fig. S6J), indicating the specific inhibitory effect of lipid peroxidation end products on the cGAS-STING pathway in COAD. Furthermore, luciferase assays demonstrated that pretreatment with 4-HNE (removed after 4-hr incubation) did not suppress STING-induced *IFNB* luciferase activity, whereas treatment with 4-HNE after STING transfection significantly inhibited STING-triggered signaling in HEK293T cells (Fig. 5H). Immunofluorescence results showed that 4-HNE co-localized with STING in SW480 and HT29 cells, suggesting that it may directly target STING and affect its properties (Fig. 5I). These results further confirm that intracellular redox homeostasis maintained by GPX4 is critical for activating the cGAS-STING signaling pathway in COAD.

### Reduced expression of GPX4 blunts the efficacy of PD-1 blockade in vivo

**Fig. 6:**
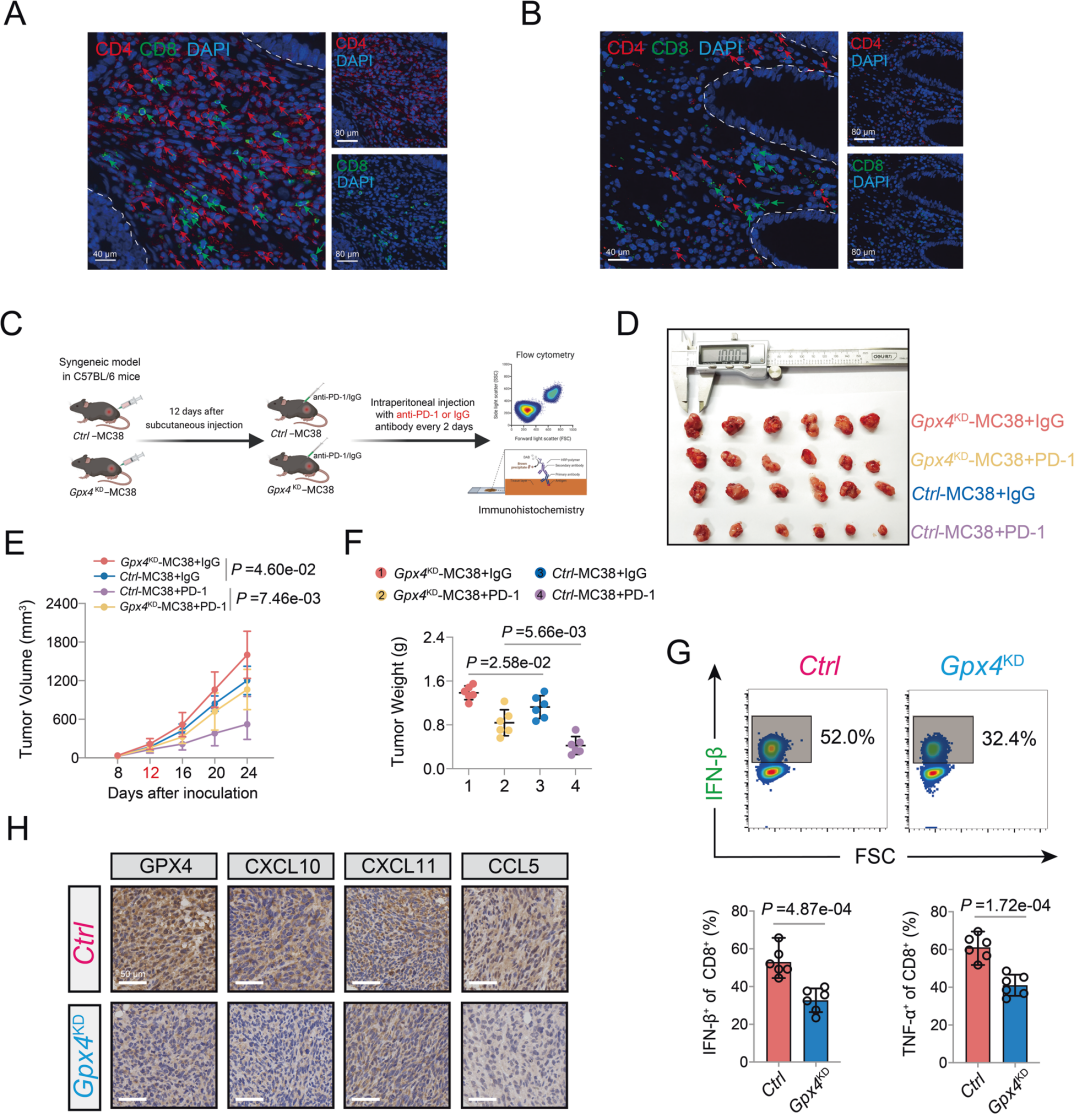
High expression of GPX4 is essential for responsiveness to PD-1blockade in COAD. **A** Representative co-staining images of GPX4, CD4, and CD8 in the high-GPX4 expression patient with COAD. **B** Representative co-staining images of GPX4, CD4, and CD8 in the low-GPX4 expression patient with COAD. **C** Schematic diagram showing the effect of *Gpx4* knockdown on MC38 tumor growth under PD-1/IgG treatment in syngeneic C57BL/6 mice (*n* = 6 mice per group). **D** Images of the dissected subcutaneous tumors from the tumor-bearing mice at the end of experiment (*n* = 6 mice per group). **E** Time-course evaluation of MC38 tumor volumes measured every 4 days in C57BL/6 mice. **F** The final weight of the MC38 tumor was shown in the scatter plot for indicated groups. **G** Representative images and statistical quantitation of the FACS analysis of the percentage of IFN-β^+^ CD8^+^ and TNF-α^+^ CD8^+^ TILs from *Ctrl*-MC38 or *Gpx4*^KD^-MC38 tumors. **H** Representative photographs indicating the expression of GPX4, CXCL10, CXCL11, and CCL5 in *Ctrl*-MC38 or *Gpx4*^KD^-MC38 tumors.

Based on the results of the in vitro data mentioned above, we next investigated whether GPX4 influences the immune response in vivo. In agreement with the bioinformatic analysis, we found that the COAD samples with high expression level of GPX4 presented higher infiltration levels of CD4 + and CD8 + T cells (Fig. 6A and Fig. S6K). On the contrary, lower infiltration levels of CD4 and CD8 + T cells were observed in COAD samples with low expression level of GPX4 (Fig. 6B and Fig. S6K). For further confirmation, a syngeneic mouse tumor model of COAD was established (Fig. 6C). The results indicated that the tumor volume of the wild-type was lower than that of the *Gpx4*-KD group after IgG treatment, and the tumor volume of the wild-type was also significantly lower than that of the *Gpx4*-KD group after anti-PD-1 therapy, indicating a potential role of GPX4 in the responsiveness to PD-1 blockade (Fig. 6D, E). Likewise, the average weight of the tumors in control group was significantly lower than that in the *Gpx4*-KD group after treatment with PD-1 antibody (Fig. 6F). In addition, flow cytometry analysis of tumor-infiltrating immunocytes indicated significantly decreased IFN-β and TNF-α levels in the *Gpx4*-KD group (Fig. 6G). Finally, the immunohistochemistry results showed decreased staining of chemokines downstream of the endogenous STING pathway, including CXCL10, CXCL11, and CCL5 in the *Gpx4*-KD group (Fig. 6H and Fig. S6L). Together, these in vivo results demonstrated that GPX4 could promote the host antitumor immune response of COAD and enhance the efficacy of cancer immunotherapy.

### Interaction between GPX4 and m6A/m5C methylation

**Fig. 7:**
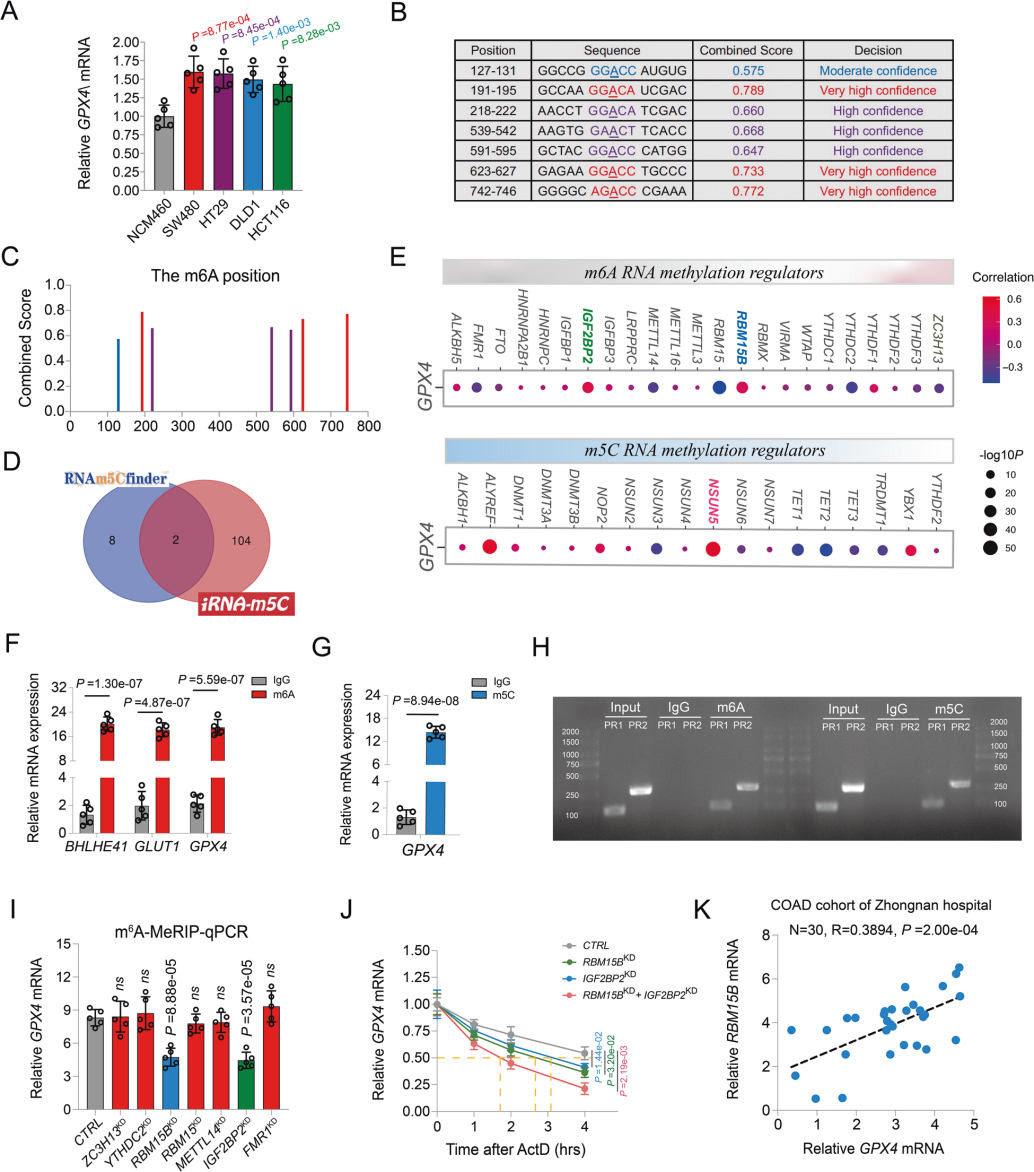
The Interaction Between GPX4 With m6A and m5C Methylation. **A** The relative *GPX4* mRNA expression level in in different COAD cell lines and normal human colonic epithelial cells. **B**, **C** Predicted m6A modification site and motif of GPX4 from *SRAMP* database. **D** Predicted m5C modification site from according to the intersection of *RNA m5C finder* and *iRNA-m5C* databases. **E** The correlation analysis of the expression level of GPX4 with that of the m6A and m5C RNA methylation regulators in TCGA-COAD. **F** MeRIP-qPCR analysis indicated that the enrichment of *BHLHE41*, *GLUT1*, and *GPX4* mRNA precipitated by m6A antibody. **G** MeRIP-qPCR analysis indicated that the enrichment of *GPX4* mRNA precipitated by m5C antibody. **H** Agarose gel analysis of MeRIP-PCR productions for the validation of m6A and m5C modification of *GPX4* mRNA. Input, IgG, m5C and m6A indicated different group from MeRIP-PCR products using different antibody. PR1 and PR2 represent different primers 1 and 2 of *GPX4* mRNA. **I** m6A-MeRIP-qPCR analysis of the expression level of *GPX4* mRNA after knocking down the indicated m6A regulators. (**J**) The time course of *GPX4* mRNA degradation with different transfections after ActD treatment in SW480 cells. **K** The correlation between relative *GPX4* mRNA expression level and *RBM15B* mRNA in the COAD cohort of Zhongnan hospital.

**Fig. 8:**
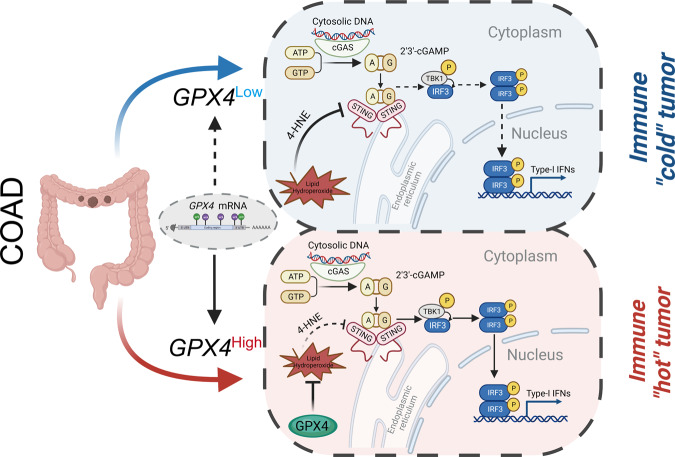
Model showing that m6A and m5C modification of GPX4 regulates cancer immunotherapy through STING activation in COAD.

The exact cause underlying high GPX4 expression in COAD remains unclear. Interestingly, we found that the expression levels of *GPX4* heterogeneous nuclear RNA (hnRNA) did not differ between normal human colonic epithelial cells and COAD cells, whereas higher expression of *GPX4* mRNA level were observed in COAD cells than normal human colonic epithelial cells (Fig. 7A and Fig. S7A). Therefore, we speculate that this discrepancy in *GPX4* mRNA levels may be the result of post-transcriptional modifications. m6A and m5C are the most common epigenetic modifications. Using SRAMP tools, a sequence-based database of m6A modification site predictors, seven potential m6A sites in *GPX4* mRNA were identified, of which three were very high confidence m6A modification sites (Fig. 7B, C). The secondary structures of the m6A-modified *GPX4* mRNA, predicted with very high confidence, are shown in Fig. S7B–D. Using the RNA m5C finder database and the iRNA-m5C tools, we identified two highly reliable m5C modification sites in the *GPX4* transcript (Fig. 7D). Several m6A and m5C regulators were differently expressed among the high- and low-GPX4 subgroups (Fig. S7E, F). Moreover, correlation analysis results indicated that GPX4 expression was significantly associated with that of most m6A regulators, such as *IGFBP2*, *IGFBP3*, *RBM15B*, *YTHDC*, and *FTO*, as well as most m5C regulators, such as *NSUN3*, *NSUN5*, *NSUN6*, *TET1*, *TET2*, and *YBX1* (Fig. 7E). MeRIP-qPCR assay and PCR-agarose gel analysis results further validated the modification of the *GPX4* transcript. Consistently, the enrichment of G*PX4* mRNA was observed using both m6A- and m5C-specific antibodies (Fig. 7F, G). MeRIP-PCR and agarose gel analysis further confirmed the presence of m6A and m5C modifications in the *GPX4* mRNA using two pairs of primers (Fig. 7H). Based on these results, the m6A and m5C regulators whose correlation coefficient was greater than 0.3 ( | R | > 0.3) with a *P*-value less than 0.05 were further studied. However, the results of m6A MeRIP-qPCR analysis and m5C MeRIP-qPCR analysis indicated that *GPX4* mRNA were only significantly decreased after knockdown of the *RBM15B*, *IGFBP2*, and *NSUN5* genes (Fig. 7I and Fig. S8A). As displayed in Fig. 7J and Fig. S8B, the half-life of *GPX4* mRNA was significantly decreased after knockdown of the *RBM15B*, *IGFBP2*, and *NSUN5*, respectively. In addition, co-knockdown of *RBM15B* and the *IGFBP2* or *NSUN5* further decreased the half-life of *GPX4* mRNA (Fig. 7J and Fig. S8B). In the COAD cohort of the Zhongnan hospital, the relative expression of *GPX4* mRNA was positively correlated with the that of the *RBM15B*, *IGFBP2* and *NSUN5*, respectively, confirming these potential regulatory relationships (Fig. 7K and Fig. S8C, D). Taken together, these findings reveal new mechanisms regulating anticancer immunity, wherein m6A and m5C modifications of *GPX4* promote cGAS-STING signaling activation by maintaining redox homeostasis in COAD (Fig. 8).

## Discussion

COAD is a highly heterogeneous disease with extensive inter- and intratumor variability, which leads to diverse responses to anticancer therapeutics [8, 9]. Therefore, in clinical practice, various factors, including clinical symptoms, side effects, pathological results, immune TME status, and genetic examination, should be comprehensively evaluated before commencing cancer immunotherapy, especially for COAD. Cancer immunotherapy is arguably the fastest-advancing realm of cancer treatment in recent years and has shown tremendous promise for tumor clearance. ICIs represent some of the most efficient anticancer immunotherapeutic agents and have shown broad clinical applicability in various malignancies. PD-L1 expression status in tumor cells was the first FDA-approved diagnostic biomarker to predict the responses of ICI therapy [28, 29]. However, this is not sufficient for optimal patient selection or for improving therapeutic outcomes due to the intra‐tumor heterogeneity of PD-L1 and the labile nature of PD-L1 expression [30]. Several clinical trials have shown that some PD-L1-negative patients respond to PD-1/PD-L1 blockade, whereas few PD-L1-positive cases have been reported to be refractory to anti-PD-1 therapy [31, 32]. TMB is a potential predictive biomarker for ICIs in both MSI-H and MSS cancers, and selection based on TMB levels can select more suitable patients for ICIs or expand the candidate pool for cancer immunotherapy [33]. However, as a novel biomarker, TMB also has limitations because the current lack of standard cut-off values evidently challenges the implementation of TMB testing in the clinic and the calculation methods of TMB differ among laboratories and assays [34, 35]. Hence, perfect biomarkers that can faithfully predict the efficacy of ICIs are yet to be identified, even for clinically recognized biomarkers, including PD-L1, TMB, and MSI-H/dMMR. Global pan-cancer gene expression profiling has revealed that GPX4 is highly expressed in most cancer types, including COAD. Thus, GPX4 could be employed as an effective biomarker in COAD. Furthermore, high expression of GPX4 was significantly associated with enhanced abundance of immune cell infiltration and increased expression of immune checkpoint genes in the COAD microenvironment, corresponding to the immune-inflamed phenotype. Collectively, these integrative analyses indicate the potential involvement of GPX4 in the regulation of the immune effector process in COAD; however, the detailed regulatory mechanism remains largely unknown.

The ability to directly reduce phospholipid hydroperoxides and oxidized lipoproteins within bio-membranes makes GPX4 unique in preventing lethal lipid oxidation [12]. Studies have indicated that GPX4 inhibitors strongly enhance the activation of T cells in the tumor microenvironment when combined with immunotherapy, which could provide novel insights into precision medicine for LAR-TNBC [13]. cGAS-STING-mediated type I interferon responses inhibit the proliferation of cancer cells, regulate cell chemosensitivity, and are involved in cancer immunoediting [21, 24, 36]. GPX4 is required for the activation of cGAS-STING pathway in innate immune responses against DNA viruses [25]. Mechanistically, GPX4 deficiency enhances lipid peroxidation and cellular level of 4-HNE induced by viral infection, thereby promoting STING carbonylation and inhibiting its activation [25]. This study demonstrates that GPX4 is essential for activating cGAS-STING signaling pathway by inhibiting the peroxidation of cell membrane lipids in COAD. These findings provide novel mechanistic insights related to GPX4-mediated modulation of cGAS-STING signaling in COAD.

RNA methylation, wherein reversible post-transcriptional RNA modifications modulate biological functions, has emerged as an important epigenetic mechanism for regulating gene expression [37]. RNA methylation regulates most aspects of RNA metabolism, including processing, transport, translation, and decay [38]. Among these, m6A and m5C have been extensively studied in cancer research [17, 19, 39–43]. In the sepsis-associated acute lung injury model, neutrophil extracellular traps induced ferroptosis in alveolar epithelial cells via activating the METTL3-YTHDF2-mediated m6A modification of GPX4 [44]. Furthermore, METTL16 enhanced GPX4 expression in a m6A-dependent manner, thus promoting the malignant progression of breast cancer via inhibiting ferroptosis [45]. RBM15B as a component of the methyltransferase complex, and can bind the METTL3 and WTAP proteins, recruiting them to specific sites in RNA [46]. As a famous m6A “reader”, IGF2BP2 was confirmed to enhance mRNA stability and translation through its recognition of m6A modifications, and involve in the occurrence and development of various malignancies [47]. NSUN5 is one of the classical m5C methyltransferase, and it was reported to control the maternal-to-zygotic transition by regulating the translation efficiency of *MAD2L2* and *GDF9* in an m5C-dependent manner [48]. Strikingly, MeRIP-qPCR and RNA-stability assays also indicated that GPX4 was directly regulated by RBM15B- and IGFBP2*-*mediated m6A modification and NSUN5-mediated m5C modification. Therefore, we speculate that the aberrant GPX4 expression may be partially attributed to m6A or m5C modifications. Nevertheless, the specific RNA methylation sites require further exploration in future studies.

## Conclusion

Taken together, our findings indicate that GPX4 may be a novel modulator of cancer immunotherapy via activating the cGAS-STING signaling pathway in COAD. Accumulating evidence has demonstrated the pivotal role of STING in the antitumor immune response; therefore, regulating STING activation holds great potential for therapeutic intervention in cancer immunotherapy. It is gratifying that the current receptor agonist amido benzimidazole exhibits potent antineoplastic activity in an immunocompetent mouse model of colon cancer [49]. However, this study has limitations, and the specific mechanism by which lipid peroxidation directly affects STING requires in-depth investigation in the future.

### Supplementary information

Supplementary Figures

Supplementary Tables

Original Data File

aj-checklist

## Data Availability

The authors confirm that the data supporting the findings of this study are available within the article or its supplementary materials. Please contact the corresponding author for any further data request or supplementary information.
