# Supplementary material for: m6A and m5C modification of GPX4 facilitates anticancer immunity via STING activation
Source: Cell Death Dis. 2023 Dec 8;14(12):809. doi: 10.1038/s41419-023-06241-w (PMC10709592; doi:10.1038/s41419-023-06241-w)
Supplement: Supplementary file 1 — Supplementary Figures [file 41419_2023_6241_MOESM1_ESM.docx]

**m6A and m5C modification of GPX4 facilitates anticancer immunity via STING activation**

Baoxiang Chen^1,2,3,4,5^, Yuntian Hong^1,2,3,5^, Xiang Zhai^1,2,3,5^, Yanrong Deng^1,2,3,5^, Heng Hu^1,2,3^, Shunhua Tian^1,2,3^, Yukang Zhang ^1,2,3^, Xianghai Ren^1,2,3,*^, Jianhong Zhao^1,2,3,*^, Congqing Jiang^1,2,3,*^

^1^Department of Colorectal and Anal Surgery, Zhongnan Hospital of Wuhan University, Wuhan 430071, China

^2^Hubei Key Laboratory of Intestinal and Colorectal Diseases, Zhongnan Hospital of Wuhan University, Wuhan 430071, China

^3^Clinical Center of Intestinal and Colorectal Diseases of Hubei Province, Zhongnan Hospital of Wuhan University, Wuhan 430071, China

^4^Rosalind & Morris Goodman Cancer Institute, McGill University, Montreal, QC, H3G 0B1, Canada

^5^These authors contributed equally to this manuscript.

*Corresponding authors: Congqing Jiang [(wb002554@whu.edu.cn),](mailto:(wb002554@whu.edu.cn),) Jianhong Zhao ([19jhzhao@whu.edu.cn](mailto:2019102040066@whu.edu.cn)) and Xianghai Ren (xhren@whu.edu.cn).

**Supplementary figures**

**
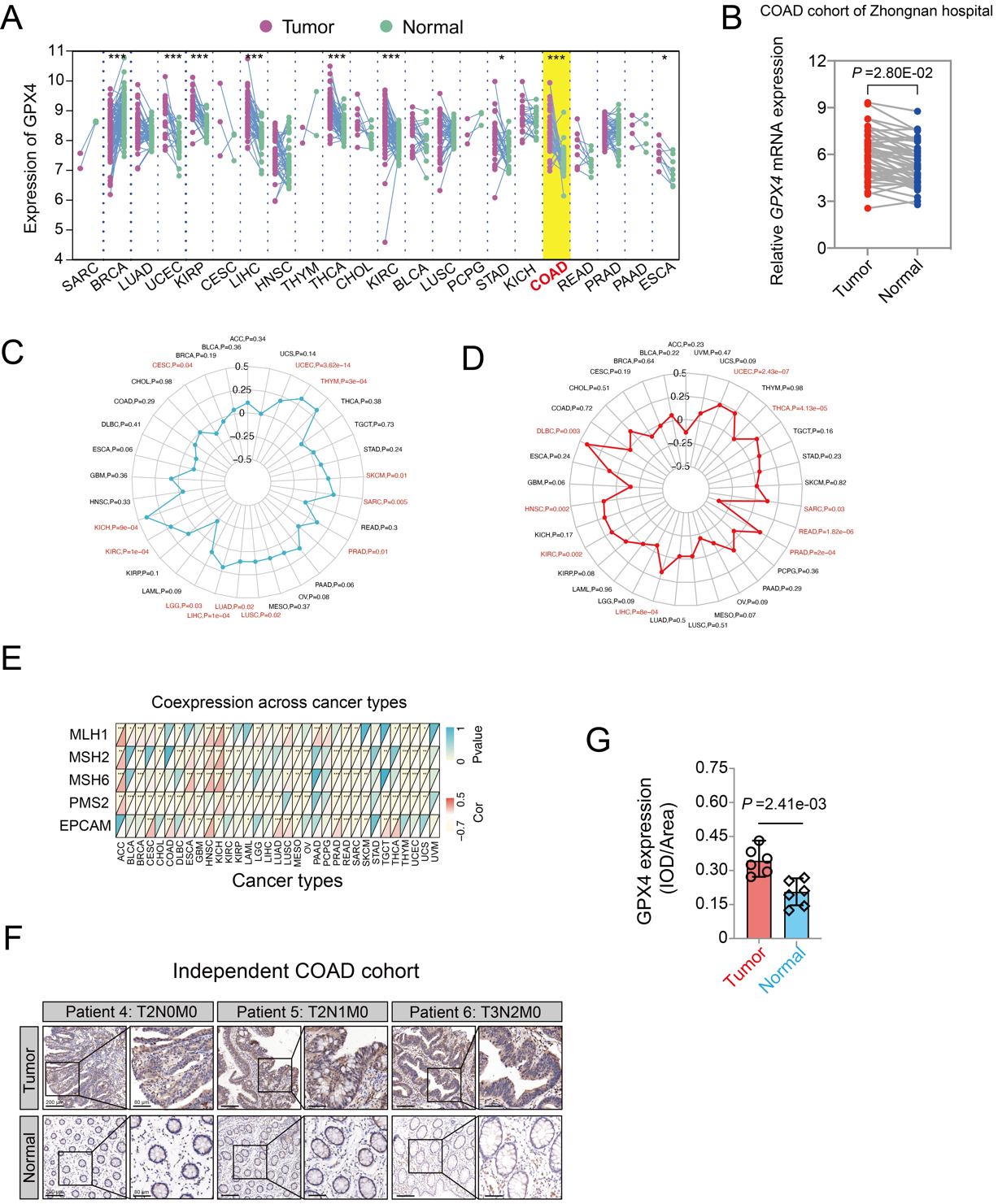
**

**Figure S1 Pan-cancer analysis of the expression, TMB and MSI of GPX4.** **A** The GPX4 expression in paired cancer tissues and adjacent normal tissues in pan-cancer using TCGA datasets. **B** RT-qPCR analysis of *GPX4* mRNA levels in 54 paired cancer tissues and adjacent normal tissues of COAD. **C** Correlation between GPX4 and TMB in pan-cancers. **D** Correlation between GPX4 and MSI in pan-cancers. **E** Heatmap visualizes the relationship of GPX4 with five DNA mismatch repair genes: MLH1, MSH2, MSH6, PMS2, and EPCAM across various cancer types. **F** Immunohistochemistry analysis of GPX4 in carcinoma tissue and para-cancer (normal) of COAD patients. **G** Quantitative data of the indicated normal or cancer tissues and the corresponding analysis which were performed by measurement of the optical density (IOD) and area and calculation of the average optical density values (IOD/Area). *, *P* < 0.05; * *, *P* < 0.01; * * *, *P* < 0.001.

**
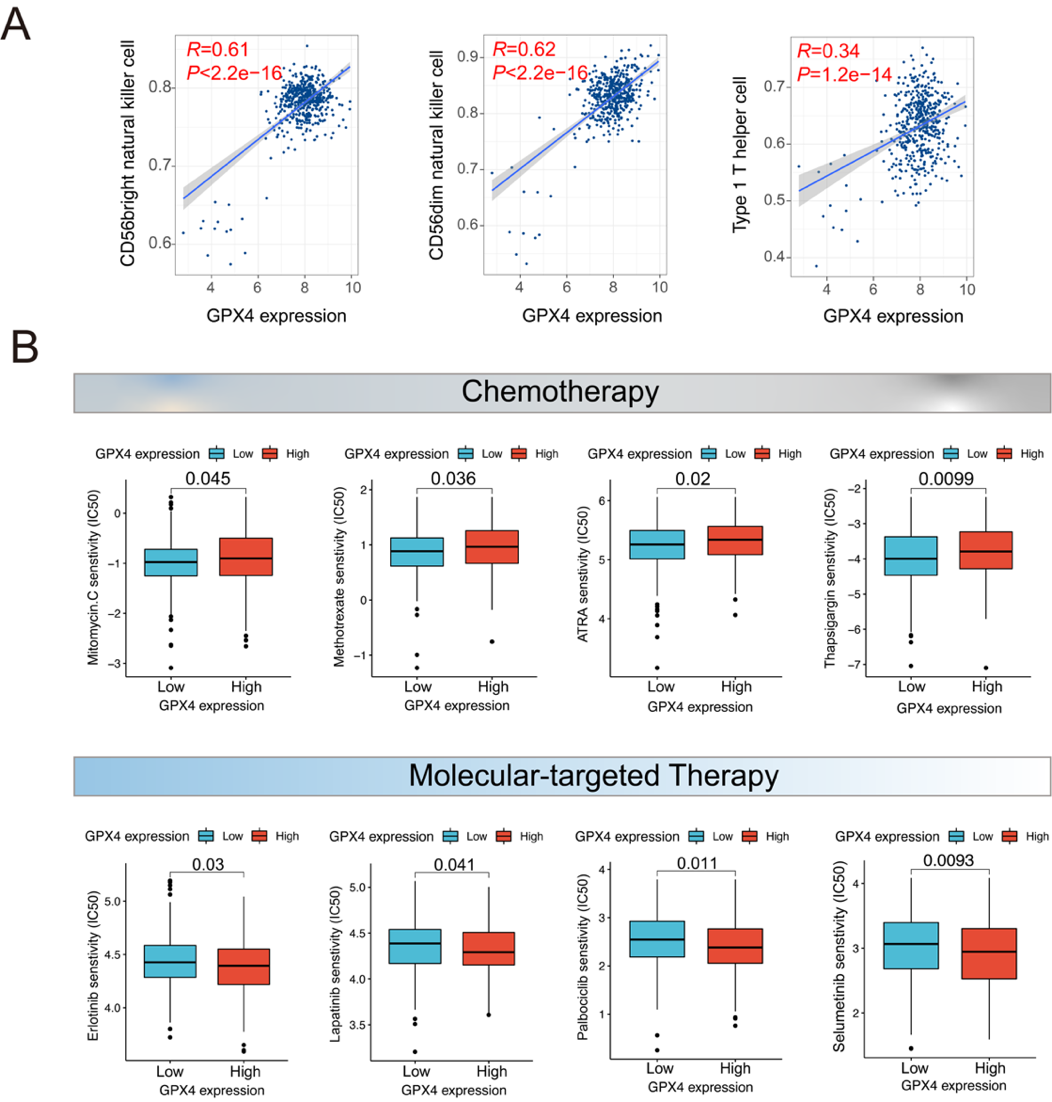
**

**Figure S2 The correlation analysis between immune response and drug sensitivity with GPX4 expression. A** The correlation analysis between GPX4 expression and immune cell infiltration in COAD. **B** The correlation analysis between GPX4 expression and several common chemotherapy drugs and molecular-targeted agents.

**
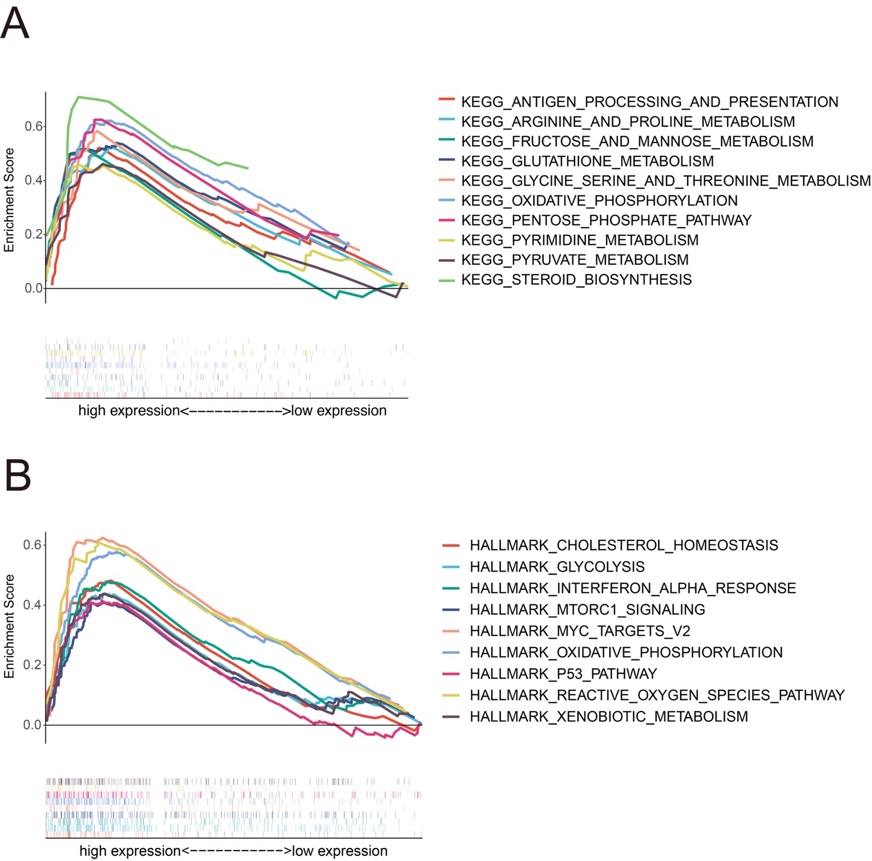
**

**Figure S3 A-B** The enrichment of KEGG and hallmark pathways in GPX4-high subpopulation.


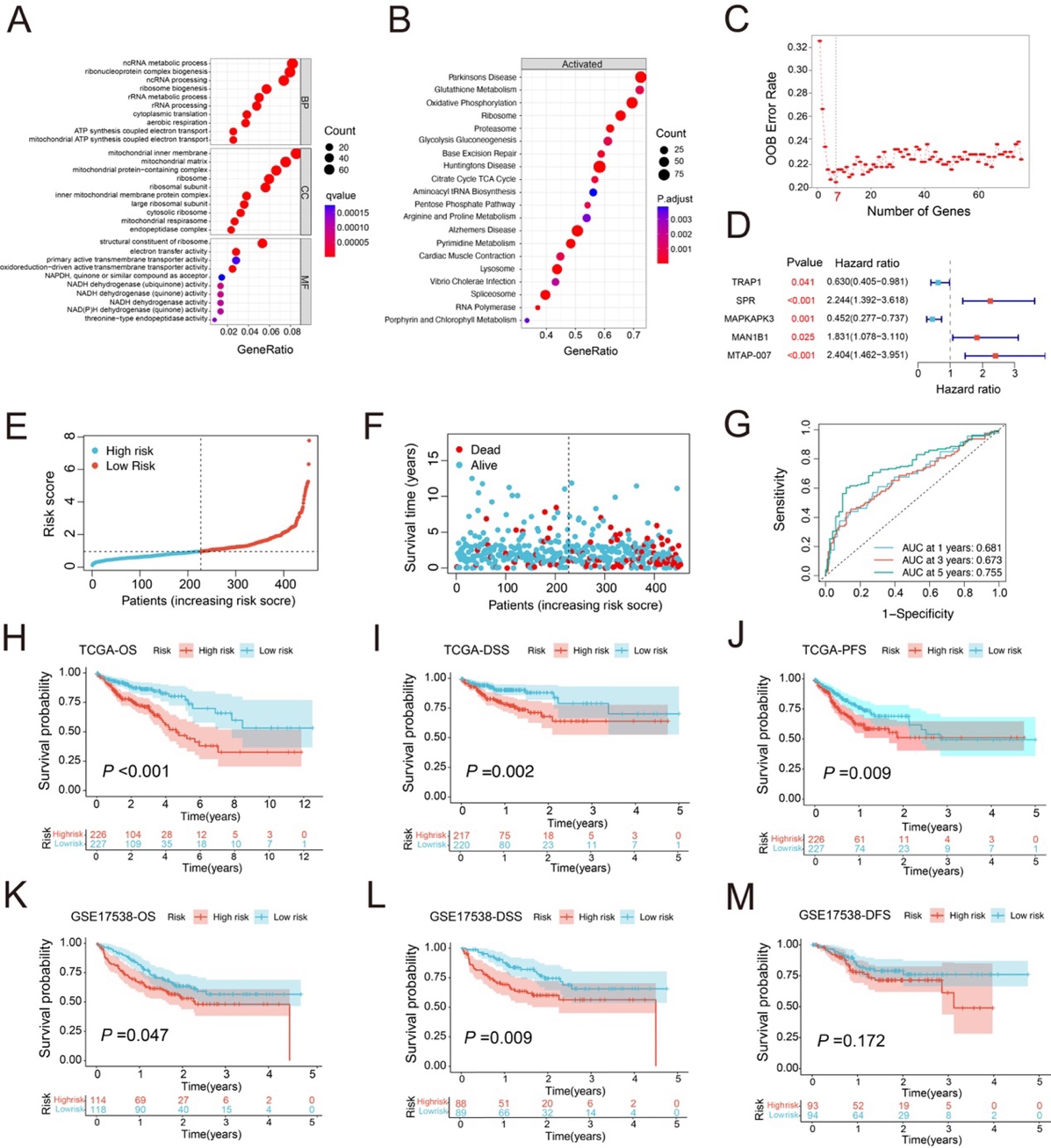


**Figure S4 Construction and Verification of the GPX4-Derived Genomic Model. A-B** GO (**A**) and KEGG (**B**) enrichment analyses of GPX4-derived genes. **C** Random survival forest indicating the relative importance of GPX4-derived genes. **D** Forest plot based on univariate Cox regression results indicates the interaction of GPX4- derived genes with COAD clinical outcomes. **E** Ranked dot showing the risk score distribution based on the GPX4- derived genes. **F** Scatter plots indicating the survival status of COAD patients. **G** ROC curves for predicting the sensitivity and specificity of 1-, 3-, and 5-year OS in accordance with GPX4-derived risk scores. **H-J** Kaplan–Meier survival curves of OS (H), DSS (I) and PFS (J) for high and low-risk scores subgroups of the TCGA-COAD cohort. **K-M** Kaplan–Meier survival curves of OS (K), DSS (L) and PFS (M) for high and low-risk scores subgroups in GSE1753 cohort.


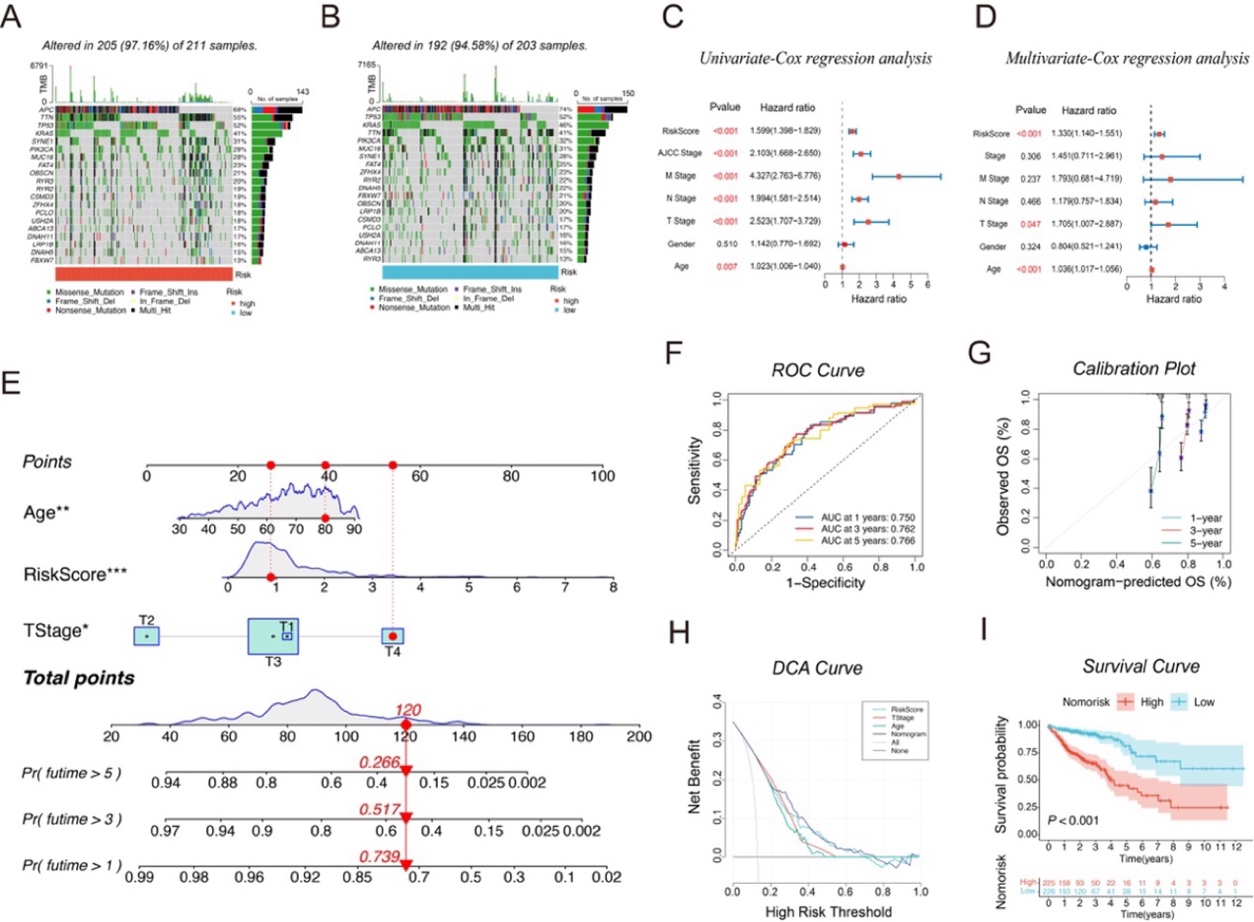


**Figure S5 Establishment and Validation of the GPX4-Derived Nomogram. A-B** Waterfall chart depicting the somatic mutation landscapes in the high (A, red) and low (B, blue) GPX4-derived risk subgroups. **C-D** Forest plots of univariate (C) and multivariate (D) Cox- regression analysis of GPX4-derived risk scores and clinical features. **E** The prognostic nomogram based on GPX4-dervied risk scores for predicting 1-, 3-, and 5-year OS. **F** ROC curves showing the predictive efficacy of 1-, 3-, and 5-year OS based on nomogram scores. **G** The calibration plots of the nomogram for predicting the 1-, 3- and 5-year OS. **H** DCA curves for evaluation of the clinical utility of the GPX4-derived nomogram. **I** Survival curves showing the results of the COAD patients based on nomogram scores.

**
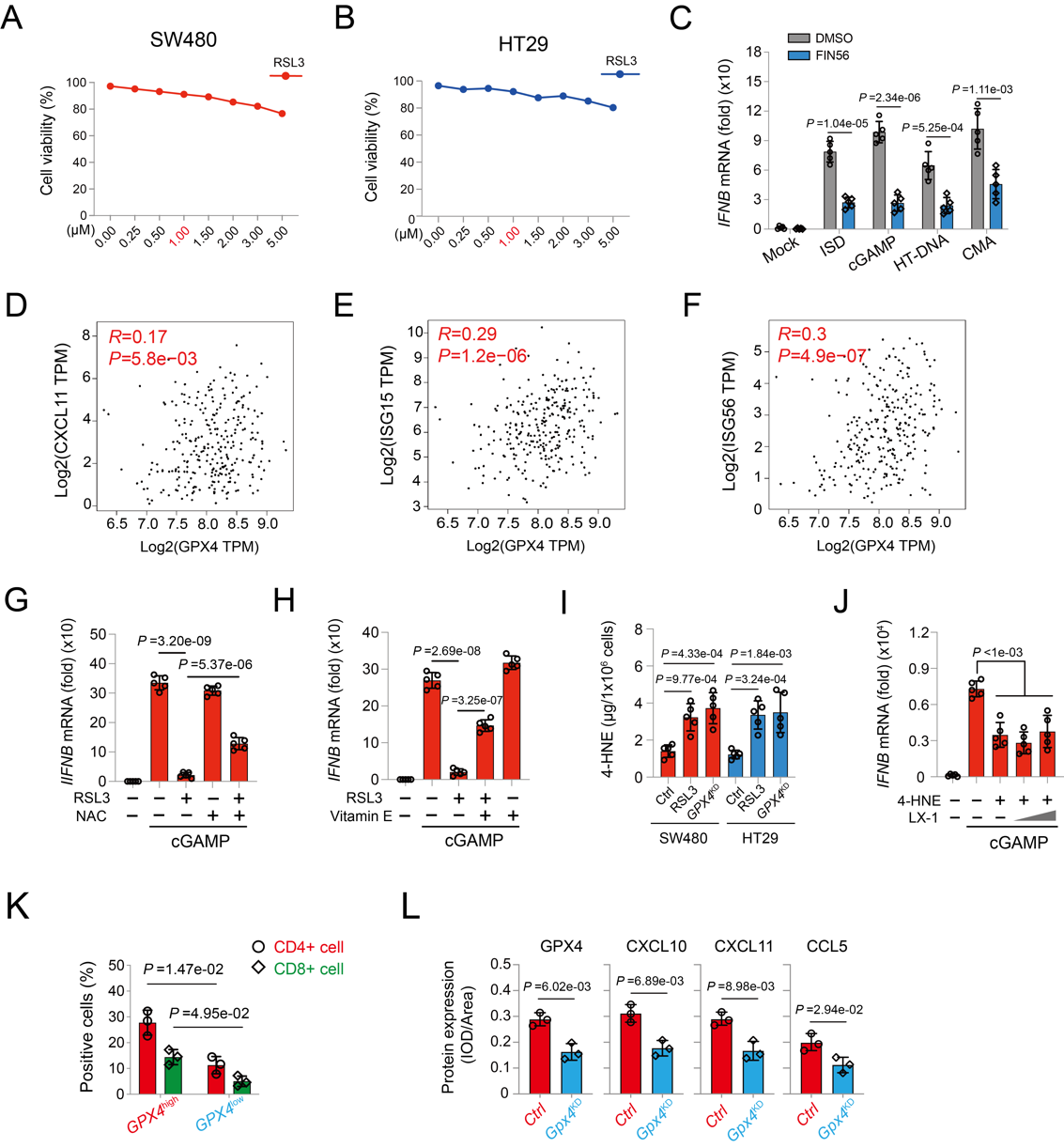
**

**Figure S6 The positive regulation of GPX4 in the cGAS–STING pathway. A-B** Cell viability analysis of SW480 and HT29 cells treated with indicated doses of RSL3**. C** RT-qPCR analysis of the *IFNB* mRNA level in SW480 cells pretreated with DMSO or FIN56 and plus stimulation as indicated. **D-F** Correlation analysis of the expression of *GPX4* level with the *CXCL11*, *ISG15* and *ISG56* expression using the *GEPIA* tool in TCGA-COAD. **G** RT-qPCR analysis of *IFNB* mRNA level in SW480 cells with NAC and RSL3, followed by cGAMP treatment. **H** RT-qPCR analysis of *IFNB* mRNA level in SW480 cells with Vitamin E and RSL3, followed by cGAMP treatment. **I** Detection of 4-HNE level in CRC cells following treatment with RSL3 or upon *GPX4* kncokdown. **J** RT-qPCR analysis of *IFNB* mRNA level in CRC cells pretreated with increasing concentrations of LX-1 and then treated with 4-HNE. **K** Quantitative analysis of CD4+ and CD8+ cells in patients with high and low GPX4 expression. **L** Quantitative analysis of the indicated tissues and the corresponding analysis which were performed by measurement of the optical density (IOD) and area and calculation of the average optical density values (IOD/Area).


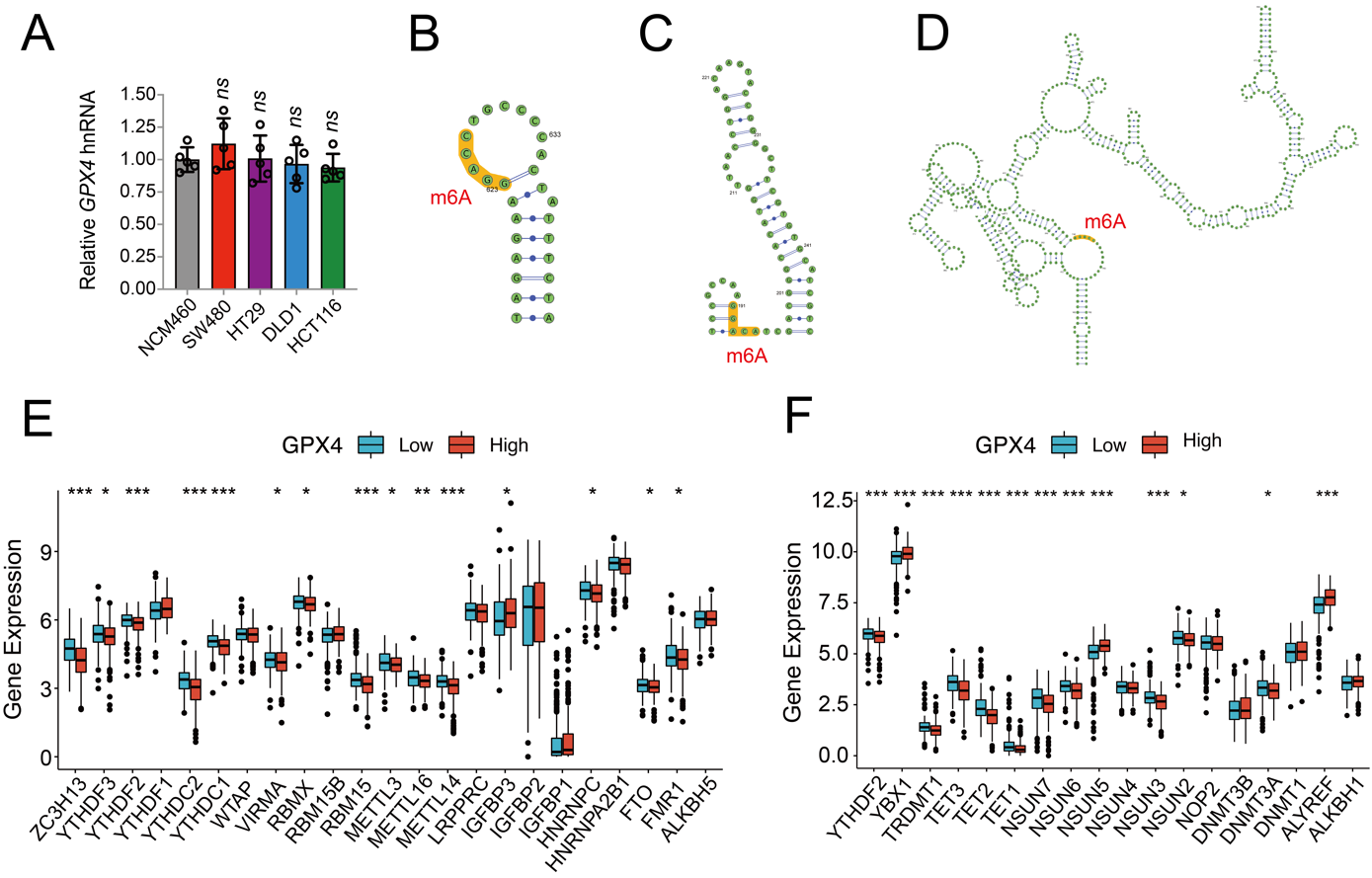


**Figure S7 The Interaction Between GPX4 With m6A and m5C Methylation. A** The relative *GPX4* hnRNA expression level in in different COAD cell lines and normal human colonic epithelial cells. **B-D** The secondary RNA structures of very high confident m6A modified GPX4. **E** Box plots showing depict the difference in expression of m6A regulators in high and low GPX4 expression subgroups across COAD specimens. **F** Box plots showing the difference in expression of m5C regulators in high and low GPX4 expression subgroups across COAD specimens.


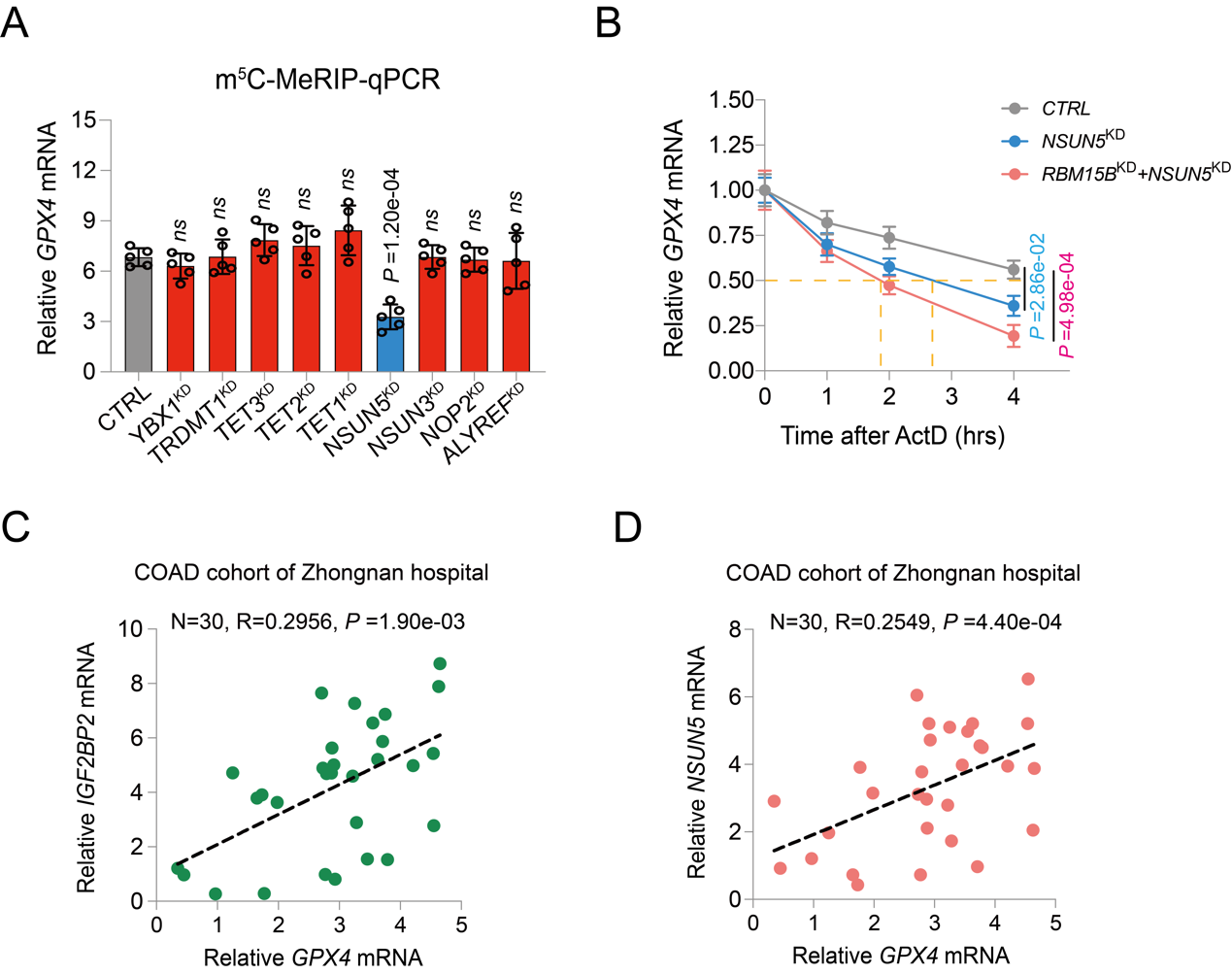


**Figure S8 The Interaction Between GPX4 With m6A and m5C Methylation. A** The m5C-MeRIP-qPCR analysis of the expression level of *GPX4* mRNA after knocking down the indicated m5C regulators. **B** The time course of *GPX4* mRNA degradation with different transfections after ActD treatment in SW480 cells. **C** The correlation between relative *GPX4* mRNA expression level and *IGF2BP2* mRNA in the COAD cohort of Zhongnan hospital. **D** The correlation between relative *GPX4* mRNA expression level and *NSUN5* mRNA in the COAD cohort of Zhongnan hospital.
