# Supplementary material for: m6A and m5C modification of GPX4 facilitates anticancer immunity via STING activation
Source: Cell Death Dis. 2023 Dec 8;14(12):809. doi: 10.1038/s41419-023-06241-w (PMC10709592; doi:10.1038/s41419-023-06241-w)
Supplement: Supplementary file 2 — Supplementary Tables [file 41419_2023_6241_MOESM2_ESM.docx]

| **Table S1.**. **All abbreviations of cancer types used in this study.** |  |
| --- | --- |
|  |  |
| **Cancer Type** | |
| **Description** | **Abbreviation** |
| Adrenocortical Carcinoma | ACC |
| Bladder Urothelial Carcinoma | BLCA |
| Breast Invasive Carcinoma | BRCA |
| Cervical Squamous Cell Carcinoma and Endocervical Adenocarcinoma | CESC |
| Cholangiocarcinoma | CHOL |
| Colon Adenocarcinoma | COAD |
| Lymphoid Neoplasm Diffuse Large B-cell Lymphoma | DLBC |
| Esophageal Carcinoma | ESCA |
| Glioblastoma Multiforme | GBM |
| Head and Neck Squamous Cell Carcinoma | HNSC |
| Kidney Chromophobe | KICH |
| Kidney Renal Clear Cell Carcinoma | KIRC |
| Kidney Renal Papillary Cell Carcinoma | KIRP |
| Acute Myeloid Leukemia | LAML |
| Brain Lower Grade Glioma | LGG |
| Liver Hepatocellular Carcinoma | LIHC |
| Lung Adenocarcinoma | LUAD |
| Lung Squamous Cell Carcinoma | LUSC |
| Mesothelioma | MESO |
| Ovarian Serous Cystadenocarcinoma | OV |
| Pancreatic Adenocarcinoma | PAAD |
| Pheochromocytoma and Paraganglioma | PCPG |
| Prostate Adenocarcinoma | PRAD |
| Rectum Adenocarcinoma | READ |
| Sarcoma | SARC |
| Skin Cutaneous Melanoma | SKCM |
| Stomach Adenocarcinoma | STAD |
| Testicular Germ Cell Tumors | TGCT |
| Thyroid Carcinoma | THCA |
| Thymoma | THYM |
| Uterine Corpus Endometrial Carcinoma | UCEC |
| Uterine Carcinosarcoma | UCS |
| Uveal Melanoma | UVM |

**Table S2. Characteristics of the patients’ cohort collected from 54 patients**

**diagnosed COAD.**

| **No.** | **Age(y)** | **Gender** | **Tumor size**  **(diameter, cm)** | **Tumor site** | **Grade** | **TNM** | **Blood vessel invasion** |
| --- | --- | --- | --- | --- | --- | --- | --- |
| 1 | 61 | M | 6 | Colon | Low | T3N0M0 | Negative |
| 2 | 62 | F | 5 | Colon | Low | T3N1bM0 | Positive |
| 3 | 66 | F | 2.5 | Colon | Low | T3N2aM0 | Positive |
| 4 | 68 | M | 7 | Colon | Low | T4bN2bM0 | Positive |
| 5 | 66 | F | 2.9 | Colon | Low | T3N0M0 | Negative |
| 6 | 51 | M | 4 | Colon | Low | T3N0M0 | Negative |
| 7 | 64 | M | 5.5 | Colon | Low | T3N0M0 | Positive |
| 8 | 71 | F | 5 | Colon | High | T3N0M0 | Negative |
| 9 | 79 | M | 3.5 | Colon | Low | T3N0M0 | Negative |
| 10 | 56 | M | 5 | Colon | Low | T3N0M0 | Negative |
| 11 | 58 | F | 5 | Colon | High | T4aN2aM0 | Positive |
| 12 | 68 | M | 8.5 | Colon | Low | T3N0M0 | Negative |
| 13 | 52 | F | 4 | Colon | Low | T2N1bM0 | Negative |
| 14 | 59 | F | 4 | Colon | Low | T3N0M0 | Positive |
| 15 | 79 | F | 3.5 | Colon | Low | T3N0M0 | Negative |
| 16 | 51 | M | 4 | Colon | High | T4N1aM0 | Positive |
| 17 | 40 | M | 4 | Colon | Low | T4N1cM0 | Positive |
| 18 | 60 | F | 8 | Colon | Low | T3N0M0 | Negative |
| 19 | 56 | F | 5 | Colon | High | T4aN1cM0 | Positive |
| 20 | 70 | M | 3 | Colon | High | T3N1M0 | Negative |
| 21 | 40 | M | 3.2 | Colon | Low | T3N0M0 | Negative |
| 22 | 78 | F | 5 | Colon | Low | T2N0M0 | Negative |
| 23 | 66 | M | 7 | Colon | Low | T3N1M0 | Positive |
| 24 | 77 | M | 4.5 | Colon | High | T3N0M0 | Positive |
| 25 | 53 | F | 1.5 | Colon | Low | T2N0M0 | Negative |
| 26 | 58 | F | 7 | Colon | High | T3N1M0 | Negative |
| 27 | 63 | F | 6 | Colon | Low | T4N1M0 | Positive |
| 28 | 48 | M | 6 | Colon | Low | T3N0M0 | Negative |
| 29 | 54 | F | 3 | Colon | Low | T3N2M0 | Negative |
| 30 | 71 | F | 4 | Colon | Low | T2N0M0 | Negative |
| 31 | 64 | M | 3 | Colon | Low | T4N1M0 | Negative |
| 32 | 68 | F | 3.5 | Colon | Low | T3N1M0 | Positive |
| 33 | 62 | F | 4.5 | Colon | Low | T3N1aM1 | Negative |
| 34 | 52 | F | 6 | Colon | Low | T3N0M0 | Negative |
| 35 | 64 | M | 3.5 | Colon | Low | T3N1aM0 | Positive |
| 36 | 78 | M | 8 | Colon | Low | T3N0M0 | Negative |
| 37 | 88 | M | 2.5 | Colon | Low | T3N0M0 | Negative |
| 38 | 72 | M | 3 | Colon | Low | T3N1aM1 | Positive |
| 39 | 62 | F | 6 | Colon | Low | T3N2bM1 | Positive |
| 40 | 83 | F | 2 | Colon | Low | T2N0M0 | Negative |
| 41 | 49 | F | 3.5 | Colon | Low | T4bN1M1 | Negative |
| 42 | 63 | M | 3.5 | Colon | Low | T4N1M0 | Negative |
| 43 | 48 | M | 4.5 | Colon | Low | T3N0M0 | Negative |
| 44 | 55 | F | 4.5 | Colon | Low | T4bN0M1 | Negative |
| 45 | 27 | M | 4.5 | Colon | High | T4aN1aM0 | Positive |
| 46 | 42 | M | 3.5 | Colon | Low | T4aN1M0 | Positive |
| 47 | 44 | F | 3.5 | Colon | Low | T3N1M0 | Positive |
| 48 | 46 | F | 8 | Colon | Low | T3N0M0 | Negative |
| 49 | 79 | F | 2.5 | Colon | Low | T3N1M0 | Negative |
| 50 | 68 | M | 3.5 | Colon | Low | T3N0M1 | Negative |
| 51 | 83 | F | 7.5 | Colon | Low | T4bN0M0 | Negative |
| 52 | 26 | M | 6.5 | Colon | High | T3N0M0 | Negative |
| 53 | 57 | M | 2.2 | Colon | Low | T3N1bM0 | Negative |
| 54 | 48 | F | 4.5 | Colon | Low | T2N0M0 | Negative |

**Table S3. The primers used in this study.**

| *GPX4*-PR1-mRNA | Forward | 5′-AGCGAGAAATCTTGGTGGAGG- 3′ |
| --- | --- | --- |
|  | Reverse | 5′-AGGACGGCAAAAATCCTCGC- 3′ |
| *GPX4*-PR2-mRNA | Forward | 5′-TCGAAATCAGGTGAAGGTCTCC- 3′ |
|  | Reverse | 5′-CAAGTCCTCCAAGCTAGGGC- 3′ |
| *IFNB*-mRNA | Forward | 5′-ATGAGTGGTGGTTGCAGGC- 3′ |
|  | Reverse | 5′-TGACCTTTCAAATGCAGTAGATTCA-3′ |
| *CXCL10*-mRNA | Forward | 5′-ATCATCCCTGCGAGCCTATCCT-3′ |
|  | Reverse | 5′-GACCTTTTTTGGCTAAACGCTTTC-3′ |
| *CCL5*-mRNA | Forward | 5′-CACCACTCCCTGCTGCTTTG-3′ |
|  | Reverse | 5′-ACACTTGGCGGTTCCTTCG-3′ |
| *GAPDH*-mRNA | Forward | 5′- GGAGCGAGATCCCTCCAAAAT -3′ |
|  | Reverse | 5′- GGCTGTTGTCATACTTCTCATGG -3′ |
| *GPX4*-hnRNA | Forward | 5′- GTGGGCGCTCGCCTGGGGTGG -3′ |
|  | Reverse | 5′- TCACACACCTTGGCCGCCACA -3′ |

**Table S4. The primary antibodies used in this study.**

| Antibody | Supplier | Catalogue | | Host |
| --- | --- | --- | --- | --- |
| GPX4 | Proteintech | 67763-1-Ig | Mouse | |
| phospho-STING | Cell Signaling Technology | #50907 | Rabbit | |
| phospho-TBK1 | Cell Signaling Technology | #5483 | Rabbit | |
| phospho-IRF3 | Cell Signaling Technology | #4947 | Rabbit | |
| m5C | Abcam | Ab10805 | Mouse | |
| m6A | Proteintech | 68055-1-Ig | Mouse | |
| GAPDH | Proteintech | 60004-1-Ig | Mouse | |
| HA | Proteintech | 51064-2-AP | Rabbit | |
| Flag | Proteintech | 66008-4-Ig | Mouse | |
| IgG | Cell Signaling Technology | #2729S | Rabbit | |
| CXCL10 | Proteintech | 10937-1-AP | Rabbit | |
| CXCL11 | Proteintech | 10707-1-AP | Rabbit | |
| CCL5 | Proteintech | 12000-1-AP | Rabbit | |
| 488 Mouse IgG2b Isotype Control | Proteintech | CL488-65128 | Mouse | |

**Table S5. The shRNA sequences of the target genes used in this study.**

| shRNA | Sequences (5’>3’) |
| --- | --- |
| shGPX4 | GTGAGGCAAGACCGAAGTAAA |
| shZC3H13 | CCTCACAATCAGGATCATCTA |
| shYTHDC2 | CGGAAGCTAAATCGAGCCTTT |
| shRBM15B | CACATGGTGATAGTTATAGTA |
| shRBM15 | CGCGGAATACAAGACTCTGAA |
| shMETTL14 | CCATGTACTTACAAGCCGATA |
| shIGF2BP2 | AGTGAAGCTGGAAGCGCATAT |
| shFMR | GCGTTTGGAGAGATTACAAAT |
| shYBX1 | CCAGTTCAAGGCAGTAAATAT |
| shTRDMT1 | CCAAAGTCATTGCTGCGATAT |
| shTET3 | ACTCCTACCACTCCTACTATG |
| shTET2 | CCTTATAGTCAGACCATGAAA |
| shTET1 | GCAGCTAATGAAGGTCCAGAA |
| shNSUN5 | CCAGGAGGAGAATGAAGACAT |
| shNUSN3 | CAAAGTTGTGTTGGATCATTT |
| shNOP2 | GCCTTCCAGAAACAGAATGAT |
| shALYREF | CGTGGAGACAGGTGGGAAACT |
| shGpx4-Mouse | ACAGCAAGATCTGTGTAAATG |
| shCtrl | TTCTCCGAACGTGTCACGT |
