# Supplementary figures and images for: m6A and m5C modification of GPX4 facilitates anticancer immunity via STING activation
Source: Cell Death Dis. 2023 Dec 8;14(12):809. doi: 10.1038/s41419-023-06241-w (PMC10709592; doi:10.1038/s41419-023-06241-w)

Figure 4F

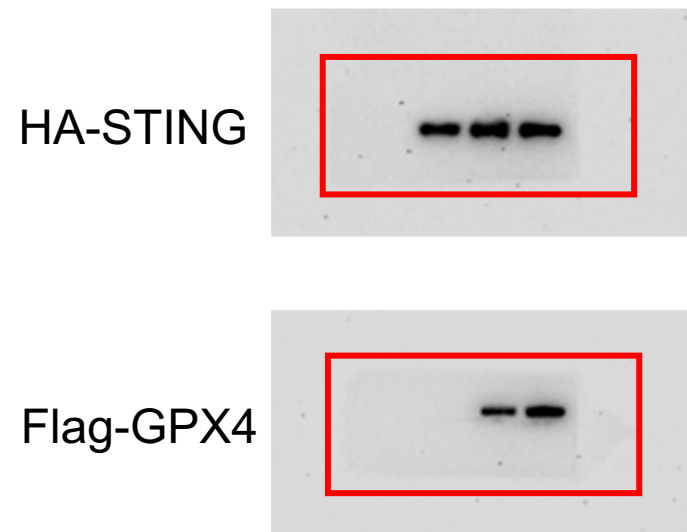

Figure 4G

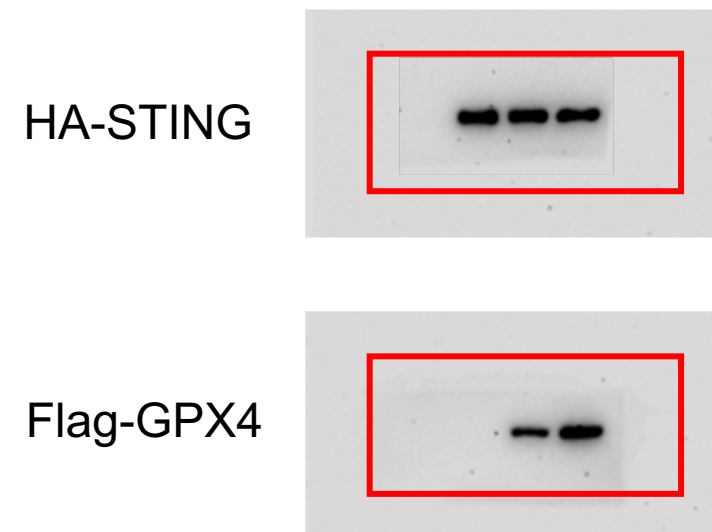

Figure 4H

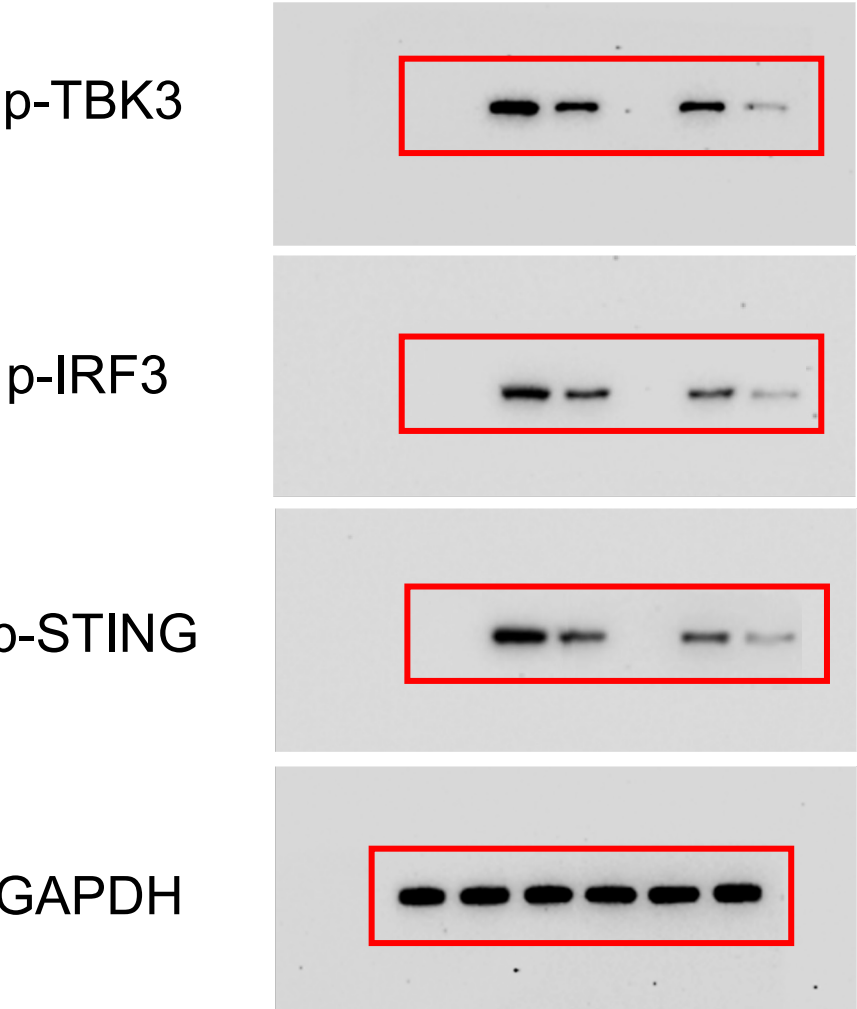

Figure 4I

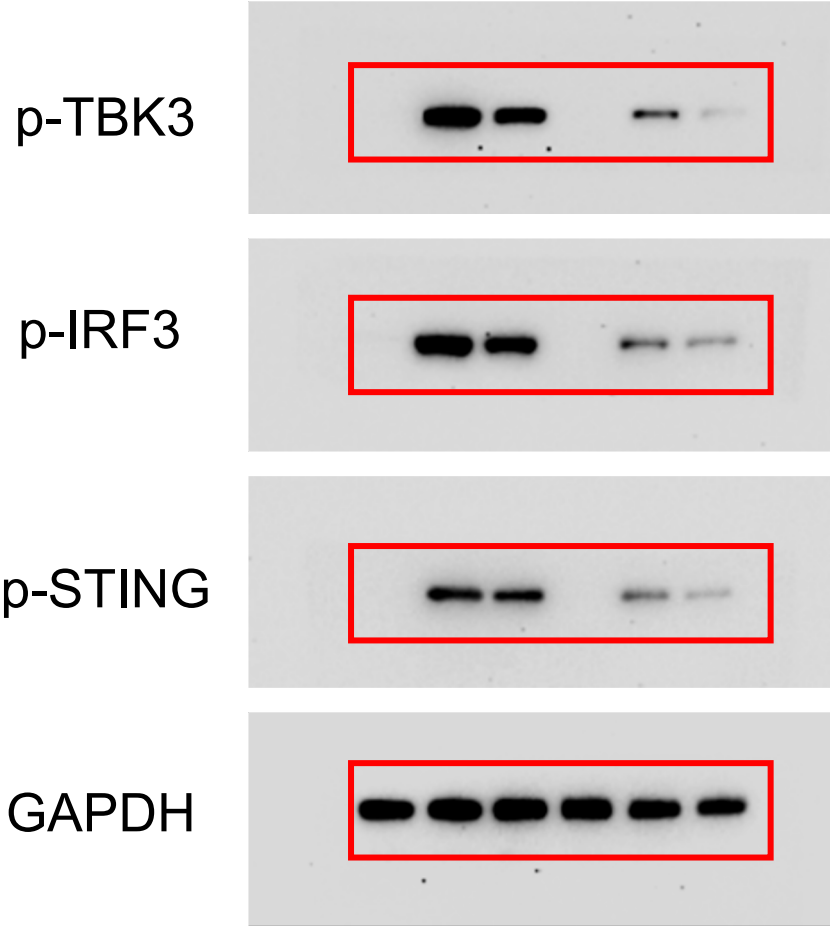

Figure 5C

p-IRF3

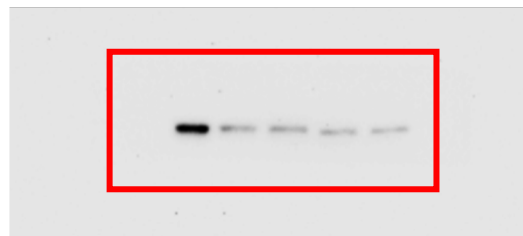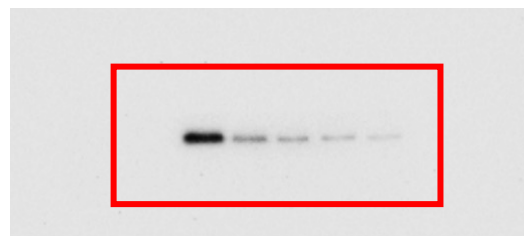

GAPDH

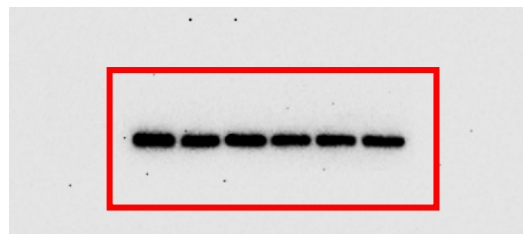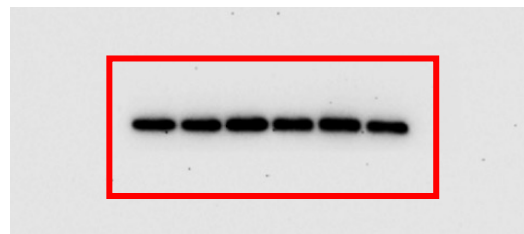

Figure 5E

p-IRF3

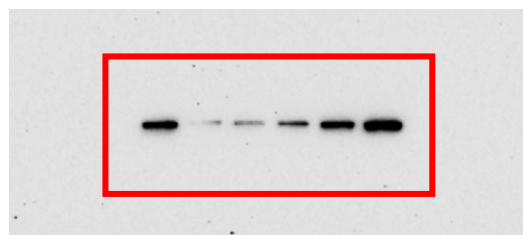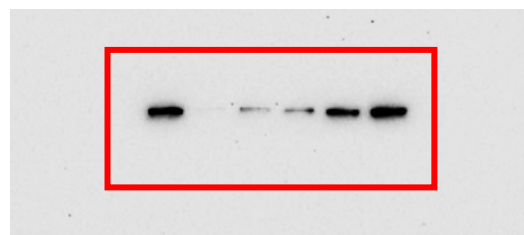

GAPDH

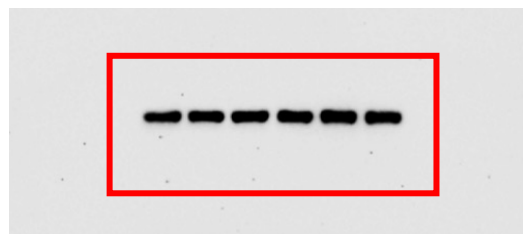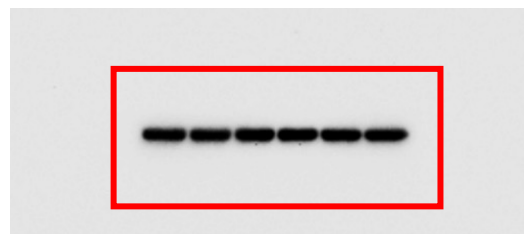

Supplement: Supplementary file 3 — Original Data File [file 41419_2023_6241_MOESM3_ESM.pdf]
